# AURKA inhibitor VIC-1911 induces mitotic defects and functional BRCAness, sensitizing prostate cancer to PARP inhibition

**DOI:** 10.1172/jci.insight.196665

**Published:** 2026-03-31

**Authors:** Galina Gritsina, Sandip Kumar Rath, Hongshun Shi, Qi Chu, Wanqing Xie, Que Thanh Thanh Nguyen, Sambhavi Senthil, Thomas J. Myers, Mehmet A. Bilen, Sarah E. Fenton, Maha Hussain, David S. Yu, Jonathan C Zhao, Jindan Yu

**Affiliations:** 1 Department of Urology and; 2 Department of Radiation Oncology, Emory University School of Medicine, Atlanta, Georgia, USA.; 3 VITRAC Therapeutics LLC, Natick, Massachusetts, USA.; 4 Department of Hematology and Medical Oncology, Emory University School of Medicine, Atlanta, Georgia, USA; 5 Winship Cancer Institute of Emory University, Atlanta, Georgia, USA.; 6 Robert H. Lurie Comprehensive Cancer Center, Northwestern University Feinberg School of Medicine, Chicago, Illinois, USA.; 7 Department of Human Genetics, Emory University School of Medicine, Atlanta, Georgia, USA.

**Keywords:** Genetics, Oncology, DNA repair, Drug therapy, Prostate cancer

## Abstract

VIC-1911 (formerly TAS-119) is a next-generation, ATP-competitive aurora kinase A (AURKA) inhibitor with a favorable biosafety profile. However, it has not been evaluated in prostate cancer (PCa), wherein AURKA is highly expressed in advanced stages and represents a critical therapeutic target. Here, we demonstrate that VIC-1911 potently inhibits AURKA activity with high selectivity over AURKB/C across diverse PCa cell lines. Treatment with VIC-1911, even at nanomolar concentrations, substantially inhibits the growth of both androgen receptor–positive (AR-positive) and AR-negative PCa cells. VIC-1911 triggers mitotic failure, induces DNA double-strand breaks (DSBs), and activates the p53 pathway, halting cell division and inducing cell death. Notably, VIC-1911 showed synergistic effects in inhibiting PCa cell growth in vitro and xenograft tumor growth in vivo with poly (ADP-ribose) polymerase inhibitors, which have proven effective in PCa with a deficiency in homologous recombination (HR) repair. Mechanistically, VIC-1911 disabled HR-mediated repair of DSBs in otherwise HR-proficient PCa cells, leading to a “BRCAness” phenotype and pronounced accumulation of DNA damage and mitotic catastrophe. In summary, our study uncovers what we believe is a novel mechanism to induce functional BRCAness through mitotic arrest and highlights VIC-1911 as a promising therapeutic agent for advanced PCa, either as a single agent or in combination, sensitizing HR-proficient tumors to PARP inhibitors.

## Abstract 2

New-generation AURKA inhibitor VIC-1911 suppresses prostate cancer growth as a single agent and further increases sensitivity to PARP inhibitors.

## Graphical abstract

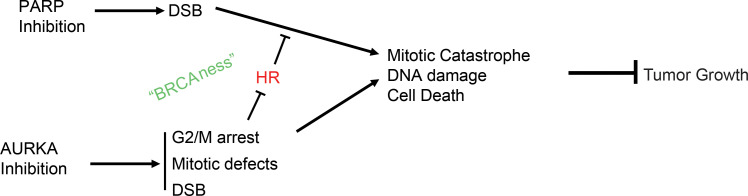

## Introduction

Prostate cancer (PCa) is the most commonly diagnosed malignancy among men in the United States and the second leading cause of cancer-related death (1). Although androgen deprivation therapy, either through surgical or chemical castration, remains the cornerstone of treatment for patients with PCa (2, 3), many patients eventually develop resistance and relapse with castration-resistant disease (4). Progression to metastatic castration-resistant prostate cancer (mCRPC) is the major cause of mortality in patients with PCa (1).

Aurora kinase A (AURKA) belongs to a family of serine/threonine kinases that play a critical role in mitosis. Specifically, AURKA phosphorylates its substrates, such as TACC3, during the G2 phase, which is an essential step for centrosome maturation during mitosis (5, 6). AURKA is frequently upregulated in aggressive forms of cancer, including mCRPC (7–9). Furthermore, longitudinal analysis of primary prostate tumor samples has revealed an increased incidence of AURKA gene amplification in a substantial subset of hormone-naive tumors that later progressed to mCRPC with neuroendocrine features (9, 10). These data suggest that AURKA amplification and/or overexpression may serve as a prognostic biomarker indicative of poor clinical outcome, and that AURKA is a major therapeutic target in late-stage PCa (7, 11). Unfortunately, a phase II clinical trial of the AURKA inhibitor alisertib in patients with mCRPC did not meet its primary endpoint, largely due to drug toxicity and the bypassing of patient preselection (12). Another phase I/II study evaluated the addition of alisertib to abiraterone in patients with mCRPC who had previously progressed on abiraterone. This study had similar limitations due to alisertib-related side effects and was terminated early, as a suitable drug dose could not be identified (13). Nevertheless, a small subset of patients still demonstrated significant clinical benefit, validating the concept of AURKA targeting in patients with advanced PCa.

VIC-1911, previously known as TAS-119, is an ATP-competitive AURKA inhibitor that binds to the catalytic domain of AURKA to inhibit its kinase activity with an IC_50_ of 1 nM (14). VIC-1911 is highly selective for binding AURKA compared with 301 other protein kinases, including AURKB (95 nM) and AURKC (36.5 nM). By contrast, alisertib showed much less selectivity, also inhibiting the kinase activities of AURKB and AURKC when used in higher doses (14). In addition, VIC-1911 targets tropomyosin receptor kinases (TRK A, B, and C) with an IC_50_ of 1.5 nM, thereby inhibiting oncogenic AKT and ERK signaling and potentially enhancing cancer cell killing. Critically, a phase I study in patients with advanced solid tumors found that VIC-1911 has a favorable safety profile distinct from other AURKA inhibitors, including alisertib (15). Most treatment-related adverse events were mild and included fatigue (32%), diarrhea (24%), and ocular toxicity (24%). Therefore, VIC-1911, as a next-generation AURKA inhibitor, may overcome the toxicity issues associated with alisertib while improving treatment efficacy in mCRPC.

In this study, we first confirmed the molecular targets of VIC-1911 in PCa cells and then examined its efficacy as a single agent in diverse PCa cell line panels. We found that VIC-1911 causes abnormal mitosis and activates DNA damage responses, consistent with the previous reports that AURKA inhibition exerts cytotoxic effects not only by inducing cell cycle arrest but also by promoting extensive DNA damage (16). We thus investigated whether VIC-1911 sensitizes PCa cells to PARP inhibitors (PARPi), which have shown high efficacy in PCa cells with mutations in *BRCA1/2* and *ATM*, and thus defective homologous recombination (HR) repair of DNA double-strand breaks (DSBs) (17–19). However, HR-proficient mCRPC is unresponsive to PARPi therapy. We found that the combination of VIC-1911 and PARPi significantly reduced PCa tumor growth, even in HR-proficient models. At the cellular level, this effect was associated with massive DNA damage, mitotic arrest, and a functional “BRCAness” phenotype induced by VIC-1911.

## Results

### VIC-1911 selectively and effectively inhibits AURKA activities.

**Figure 1:**
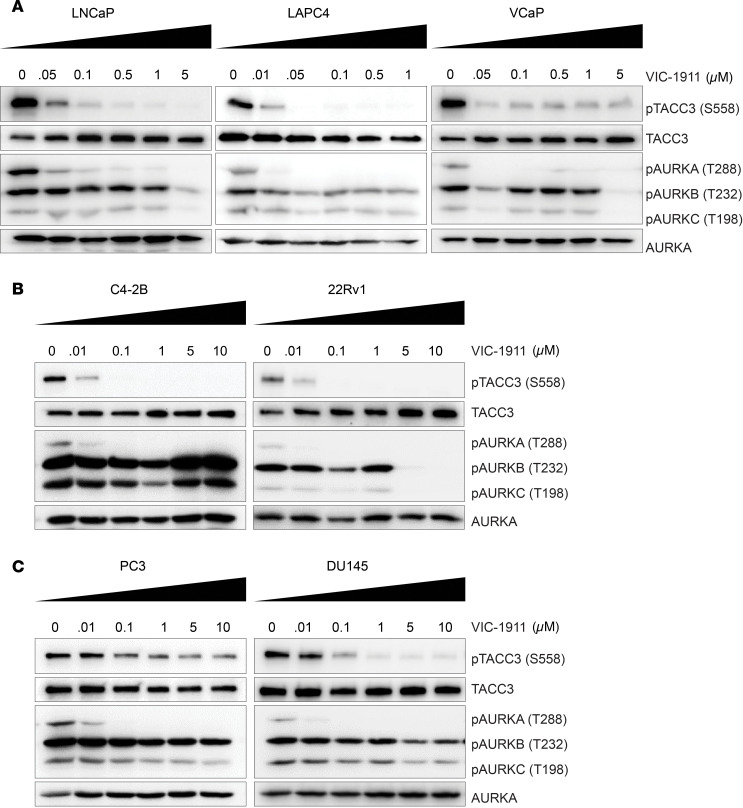
VIC-1911 robustly inhibits AURKA activities in a diverse panel of PCa cells. (**A**–**C**). Immunoblotting images show a gradual decrease in phospho-TACC3 and phospho-AURKA levels with increasing doses of VIC-1911 in AR-positive, androgen-dependent PCa cells (**A**), AR-positive, androgen-independent PCa cells (**B**), and AR-negative PCa cells (**C**). Cells were synchronized by overnight exposure to nocodazole (100 ng/mL) and cotreated with VIC-1911 for 24 hours.

To assess the on-target effects of VIC-1911 in PCa cells, we performed overnight incubation with a panel of androgen-dependent, castration-sensitive PCa cells, including LNCaP, VCaP, and LAPC4 (Figure 1A). Immunoblotting analyses revealed that VIC-1911 treatment markedly reduced phosphorylation of AURKA and TACC3, an AURKA cognate substrate (20), at concentrations as low as 0.05 μM. Critically, both AURKB and AURKC phosphorylation status remained unaffected by VIC-1911 at concentrations up to 1 μM, suggesting at least 20-fold higher selectivity for AURKA (Figure 1A). To further validate this finding, we examined androgen receptor–positive (AR-positive) but androgen-independent cell lines, 22Rv1 and C4-2B, that represent a majority of clinical CRPC patients. We found that VIC-1911 remained highly effective and selective in inhibiting AURKA activity in these CRPC cell lines (Figure 1B). Interestingly, in C4-2B cells, as low as 0.01 μM VIC-1911 was sufficient to substantially reduce AURKA activity and as high as 10 μM still failed to reduce AURKB and AURKC phosphorylation levels, indicating over 1,000 times increased selectivity for AURKA over AURKB/C. Similar high selectivity was also observed in AR-negative PC3 and DU145 cells (Figure 1C).

Further, as VIC-1911 is known to also target TRKs (14), we evaluated the effect of VIC-1911 on activation of MAPK1/2 (ERK) and AKT, 2 kinases that play an important role in CRPC progression (21, 22). We observed a dose-dependent decrease in ERK phosphorylation under VIC-1911 treatment in androgen-sensitive LNCaP cells, AR-positive but androgen-insensitive 22Rv1 cells, and AR-negative PC3 cells. In contrast, AKT phosphorylation did not show a robust response. Among the 3 tested cell lines, only 22Rv1 exhibited decreased AKT activation (Supplemental Figure 1A; supplemental material available online with this article; https://doi.org/10.1172/jci.insight.196665DS1). In aggregate, our data validate VIC-1911 as a highly selective and effective AURKA inhibitor across diverse PCa cell types and show enhanced selectivity for AURKA relative to AURKB and AURKC in PCa cells. In addition, VIC-1911 co-targets MAPK1/2, whose activation is also frequently elevated in advanced PCa, thereby providing additional therapeutic benefit.

### VIC-1911 represses the growth of a diverse panel of PCa cells.

**Figure 2:**
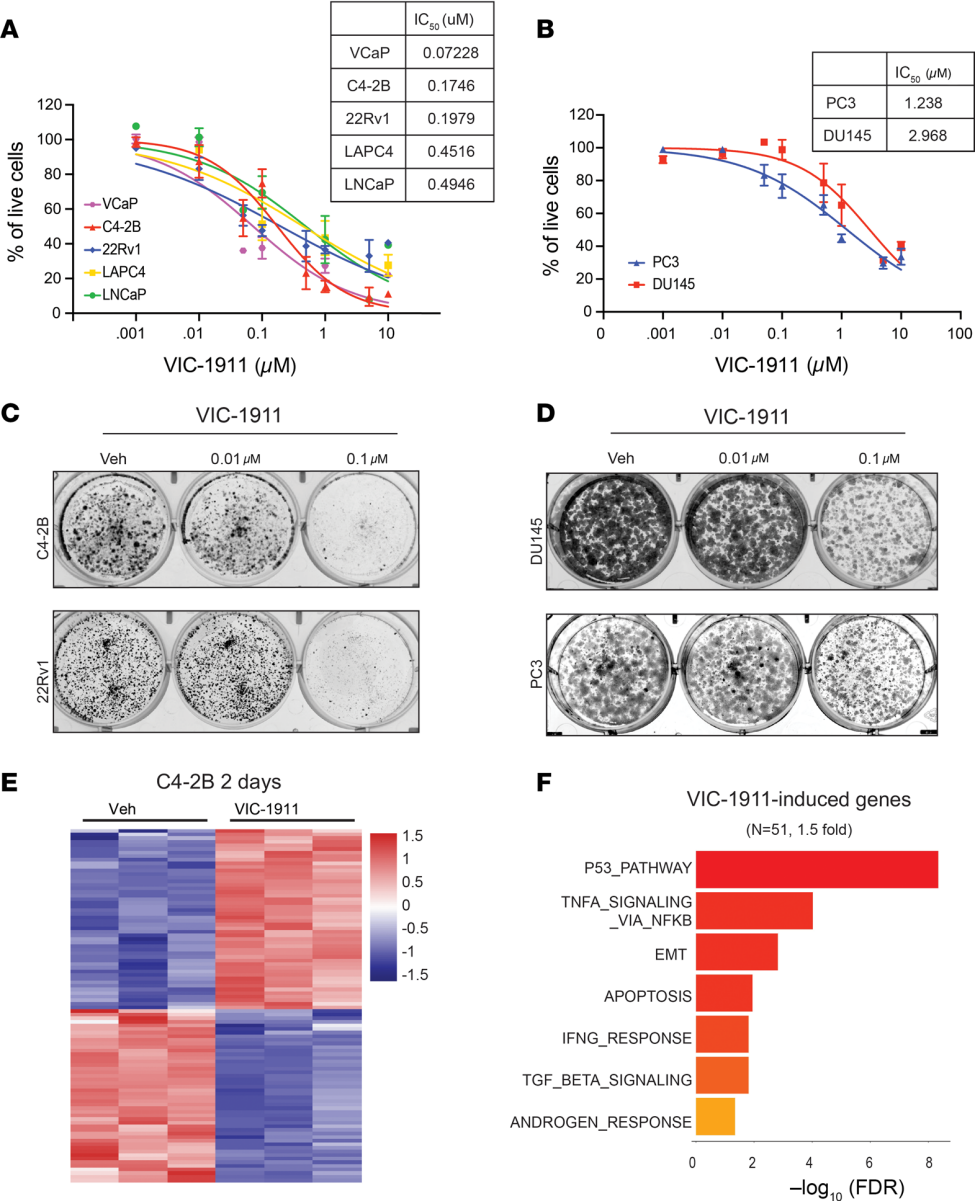
VIC-1911 inhibits cell growth and activates the P53 pathway. (**A** and **B**) Dose-response curves after 7 days of treatment with VIC-1911 of AR-positive (**A**) and AR-negative (**B**) PCa cell lines. Data are presented as mean ± SEM (*n* = 2 independent experiments). IC_50_ concentrations in PCa cell lines were calculated by the normalized response variable slope equation using GraphPad Prism 10. (**C** and **D**) Colony formation assay shows VIC-1911 inhibits PCa cell survival. A total of 6,000–10,000 cells/well of AR-positive (**C**) and AR-negative (**D**) PCa cell lines were seeded on 6-well plates and grown in the presence of different doses of VIC-1911 for 7 days, fixed, stained, and imaged. Representative experiments of *n* = 3 are presented. (**E** and **F**) C4-2B cells were subjected to triplicate RNA-Seq analyses after 48 hours of treatment with vehicle or VIC-1911 (0.1 μM). (**E**) Gene expression heatmap shows 50 upregulated and 48 repressed differentially expressed genes with 1.5-fold change. (**F**) Gene ontology analysis reveals a list of HALLMARK concepts that are induced by VIC-1911 treatment in C4-2B cells.

Given that VIC-1911 effectively inhibits AURKA across all tested PCa cell lines and considering the essential role of AURKA in regulating the cell cycle, especially mitosis, we next investigated the impact of VIC-1911 on PCa cell proliferation. The PCa cell line panel was treated with serial dilutions of VIC-1911 for 7 days, followed by cell viability analyses. Out of the AR-positive PCa cell lines, VCaP was the most sensitive, followed by C4-2B and 22Rv1, and then LNCaP and LAPC4, all of which showed an IC_50_ of less than 1 μM, suggesting that the efficacy was due to AURKA inhibition, but not AURKB and AURKC, which required high doses for inhibition (Figure 2A). Moreover, this aligns with the baseline expression and activation of AURKA in AR-positive PCa cells, with VCaP exhibiting the highest endogenous levels of total and phospho-AURKA, while the levels of phosphorylated AURKB/C remained similar across these cell lines (Supplemental Figure 2A).

Interestingly, although VIC-1911 effectively inhibited AURKA activity in PC3 and DU145 cells at concentrations as low as 0.01 μM, comparable to its effects in AR-positive cells (Figure 1C), it was less efficacious at suppressing their proliferation, likely due to AR-negative PCa cells being more resistant to G2/M cell cycle arrest, as previously noted (23, 24) (Figure 2B). Coincidentally, VIC-1911 sensitivity did not correlate with the baseline expression of AURKA in these cells, although only 2 AR-negative cell lines were tested (Supplemental Figure 2A). The IC_50_ of VIC-1911 in the AR-negative PC3 and DU145 cells was 1.2 and 2.9 μM (Figure 2B), respectively, which was still well within the range of the selectivity of VIC-1911 on AURKA compared with AURKB/C in these cells. Likewise, a colony formation assay demonstrated that 0.1 μM of VIC-1911 eliminated AR-positive C4-2B and 22Rv1 cells but only substantially slowed the growth of AR-negative PC3 and DU145 cells (Figure 2, C and D). Taken together, these data suggest that, via AURKA targeting, VIC-1911 suppresses the growth of a wide spectrum of PCa cells.

To gain insights into the mechanism by which VIC-1911 inhibits PCa cell growth, we treated C4-2B cells with 0.1% DMSO vehicle control or 0.1 μM of VIC-1911 for 48 hours, followed by RNA-Seq analysis to evaluate changes in gene expression in response to the drug. We discovered that VIC-1911 caused 1.5-fold upregulation of 50 genes and 1.5-fold downregulation of 48 genes (Figure 2E and Supplemental Table 1). Gene Ontology (GO) analyses of VIC-1911–induced genes revealed highly significant induction of the p53 pathway, which is known to become upregulated in response to DNA damage to trigger cell cycle arrest, initiate DNA repair, and/or induce cell death (25) (Figure 2F). Indeed, TNF-α signaling and apoptosis, 2 major cell death programs, were also strongly enriched. By contrast, MTORC1 signaling, a key driver in cell growth and metabolism that is known to be suppressed by p53 activation (26), was the most inhibited pathway under VIC-1911 treatment (Supplemental Figure 2B).

### VIC-1911 drastically induces mitotic defects and DNA DSBs.

**Figure 3:**
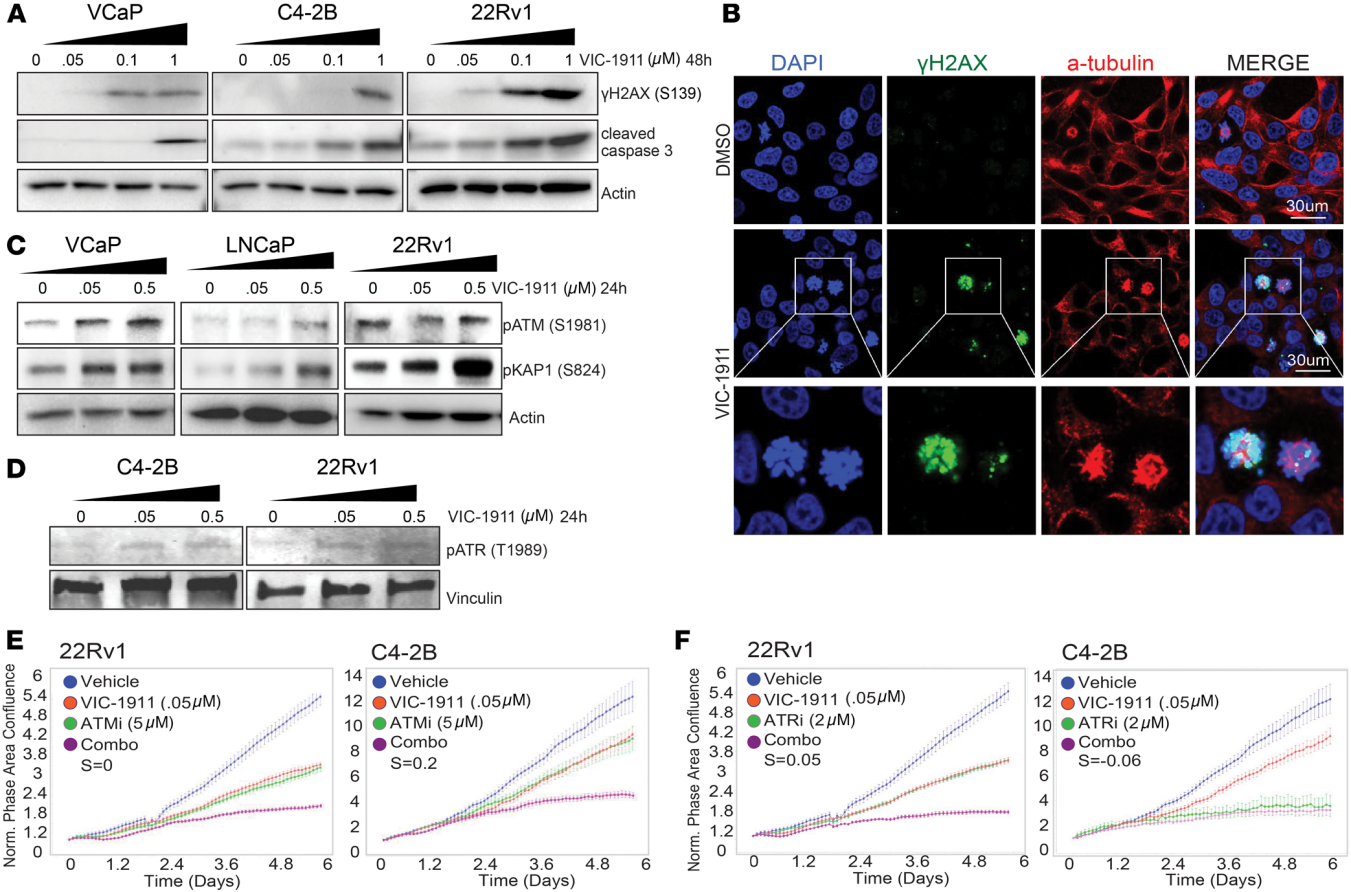
VIC-1911 induces mitotic defects, DNA damage, and apoptosis in PCa cells. (**A**) Immunoblotting analysis shows a dose-dependent increase in cleaved caspase-3 and phospho-γH2AX (S139) in response to 48 hours of incubation with increasing doses of VIC-1911. (**B**) VIC-1911 treatment induces mitotic abnormalities. Representative immunofluorescent images show increased nuclear-specific phospho-γH2AX (S139) staining in C4-2B cells with mitotic defects after 24 hours of treatment with 0.1 μM of VIC-1911. Scale bar: 30 μm. (**C**) Immunoblotting images show a gradually increasing level of phospho-ATM (S1981) in pKAP1 (S824) in response to 24 hours of treatment with increasing doses of VIC-1911. (**D**) Immunoblotting images show a gradually increasing level of phospho-ATR (T1989) in response to 24 hours of treatment with increasing doses of VIC-1911. (**E**) VIC-1911 in combination with lartesertib (ATMi) reduces the proliferation of AR-positive CRPC cells. Relative cell confluence was assessed using IncuCyte live-cell imaging. (**F**) VIC-1911 in combination with ceralasertib (ATRi) reduces the proliferation of AR-positive CRPC cells. Relativ e cell confluence was assessed using IncuCyte live-cell imaging. Bliss coefficient (S): S = 0 indicates an additive effect, S > 0 indicates synergy, and S < 0 indicates antagonism. The IncuCyte experiments were run once.

AURKA is instrumental in proper chromosome segregation during cell division, and its restriction causes G2/M checkpoint failure (27), DNA damage, mitotic arrest, and cell death (28). Thus, we aimed to investigate the immediate molecular response triggered by AURKA inhibition by VIC-1911 in PCa models. We treated PCa cell lines with increasing doses of VIC-1911. Immunoblotting revealed substantially increased γH2AX phosphorylation, a marker for DNA DSBs, suggesting that AURKA inhibition leads to major DNA damage (Figure 3A). Furthermore, VIC-1911 treatment, particularly at 1 μM, increased cleaved caspase-3 levels, indicating apoptosis (Figure 3A). Moreover, we subjected C4-2B cells to confocal imaging after 24 hours of drug treatment. Interestingly, we observed a substantial number of cells with extensive mitotic abnormalities after exposure to 0.1 μM of VIC-1911 (Figure 3B). Staining of α-tubulin revealed that some cells were arrested in the prometaphase/metaphase of mitosis, possibly due to failure in proper spindle checkpoint satisfaction, whereas other cells showed monopolar or multipolar spindles, which may have been caused by impaired centrosome separation. These cells showed a costaining of elevated γH2AX phosphorylation, indicating DSBs and DNA damage (Figure 3B). We also assessed morphological changes triggered by overnight treatment with VIC-1911 in 22Rv1 cells and observed similar induction of mitotic abnormalities and γH2AX phosphorylation (Supplemental Figure 3A). Therefore, by targeting AURKA, VIC-1911 rapidly induces mitotic defects, resulting in extensive DSBs and cell cycle arrest.

To determine whether VIC-1911 affects DNA damage response, we examined ATM and ATR signaling. Immunoblotting revealed that VIC-1911 treatment activates ATM, a critical sensor protein that detects DNA damage, such as DSBs, and becomes phosphorylated (29) (Figure 3C and Supplemental Figure 3B). Activated ATM in turn phosphorylates serine 139 of the H2A.X histone variant near the sites of DNA DSBs, which serves as a scaffold to recruit other DNA damage response proteins. Among these, KAP1, a direct ATM substrate (30), also showed robust activation upon VIC-1911 treatment (Figure 3C and Supplemental Figure 3B). These data support an active DNA damage response after VIC-1911 treatment and are consistent with the accumulation of γH2AX phosphorylation in cells with mitotic abnormalities, as shown in Figure 3B. To further examine the downstream events, we analyzed ATR, a critical DNA damage response kinase involved in S-phase and G2/M checkpoints (31) that functions as a gatekeeper for cell cycle arrest (32), and we found that VIC-1911 treatment also induced ATR activation (Figure 3D and Supplemental Figure 3C).

With this robust activation of DNA damage response regulators, we wondered whether VIC-1911 might exhibit synergistic effects with either clinically available ATM or ATR inhibitors. Interestingly, VIC-1911 and lartesertib (ATMi) showed strong combinatory effects in suppressing the growth of 22Rv1 and C4-2B cells (Figure 3E). Bliss model analysis indicated a synergistic effect in C4-2B cells but an additive effect in 22Rv1 cells at the tested dose ratio (Supplemental Table 2). The combination of VIC-1911 with ceralasertib (ATRi) showed an additive effect in 22Rv1 but not as much in C4-2B cells, which were already highly sensitive to ceralasertib alone at the tested dose (Figure 3F). These results suggest that VIC-1911 induces a DNA damage response and exhibits combinatorial effects with DNA damage–targeting agents in the treatment of PCa.

### VIC-1911 acts synergistically with PARPi to suppress PCa cell growth.

**Figure 4:**
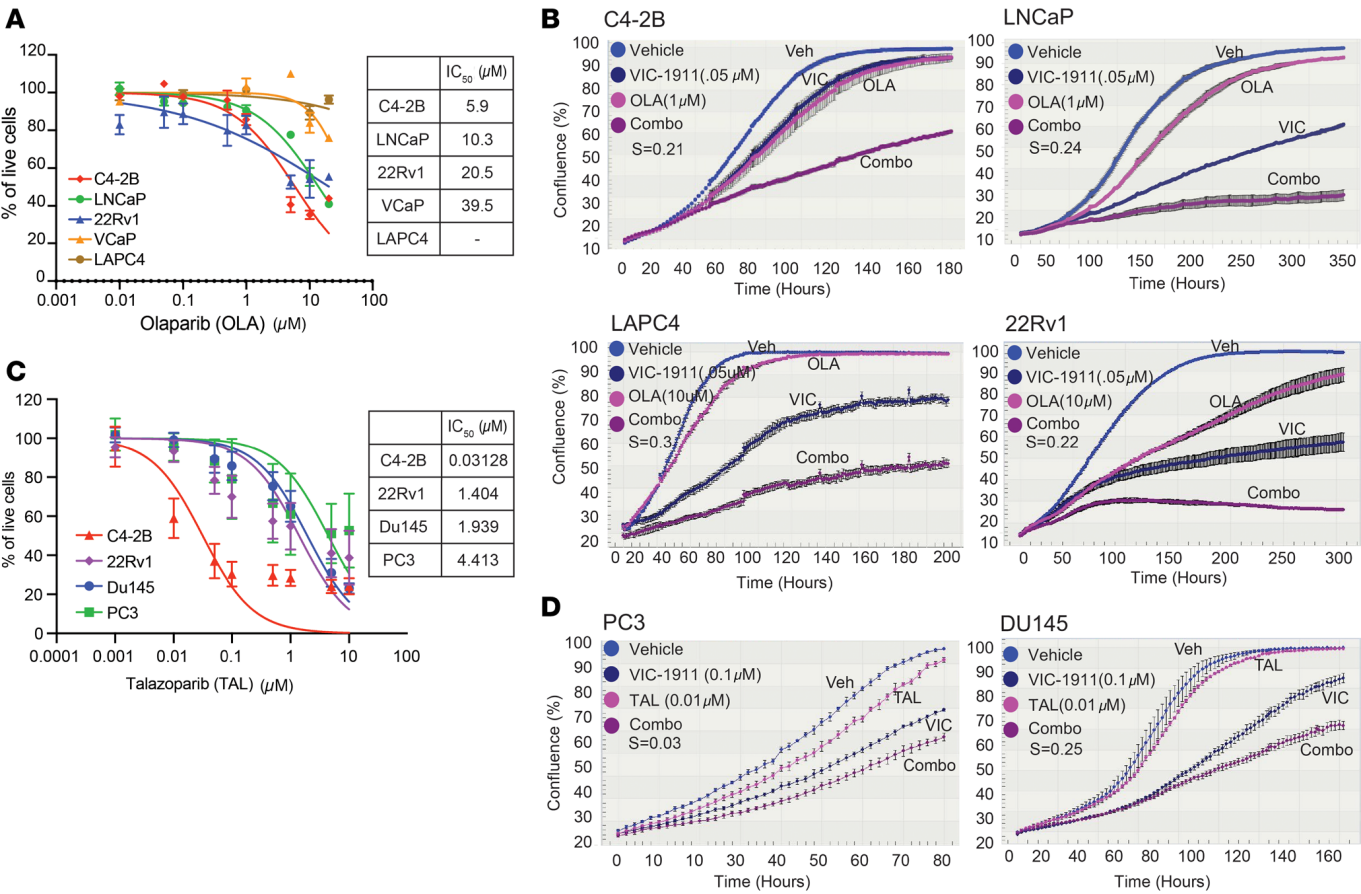
VIC-1911 acts synergistically with PARPi to inhibit PCa cell growth in vitro. (**A**) Dose-response curves after 7 days of treatment with olaparib of AR-positive PCa cell lines. Data are presented as mean ± SD (*n* = 3 independent experiments); the IC_50_ concentrations were calculated by the normalized response variable slope equation using GraphPad Prism 10. (**B**) VIC-1911 in combination with olaparib reduces the proliferation of AR-positive PCa cells. Relative cell confluence was assessed using IncuCyte live-cell imaging. The representative experiment of *n* = 3 replicates is presented. (**C**) Dose-response curves after 7 days of treatment with talazoparib of AR-positive and AR-negative CRPC cell lines. Data are presented as mean ± SD (*n* = 3 independent experiments). (**D**) VIC-1911 in combination with talazoparib reduces the proliferation of AR-negative CRPC cells. Relative cell confluence was evaluated using IncuCyte live-cell imaging. Bliss coefficient (S): S = 0 indicates an additive effect, S > 0 indicates synergy, and S < 0 indicates antagonism. The representative experiment of *n* = 3 replicates is presented.

We next hypothesized that combining PARPi with VIC-1911 could be a promising approach since VIC-1911 induces massive DNA DSBs and mitotic catastrophe. To test this hypothesis, we first treated a panel of AR-positive PCa cells with olaparib to identify optimal doses for the drug combination (Figure 4A). We found that LNCaP and C4-2B showed much better responses to olaparib than VCaP and LAPC4, being consistent with previous reports that LNCaP and C4-2B harbor some *BRCA2* and other HR gene mutations, albeit not functionally HR deficient (32). The 22Rv1 cells demonstrated much less sensitivity to olaparib than LNCaP and C4-2B but better sensitivity than VCaP and LAPC4. We thus chose 1 μM of olaparib for LNCaP and C4-2B and 10 μM for LAPC4 and 22Rv1 in combination assays. VCaP cells were not analyzed because they do not work well with the IncuCyte live-cell imaging system due to their mixed growth of adherent and clumping/floating cells. Notably, the combination of VIC-1911, used at only a 0.05 μM concentration, with olaparib substantially decreased the growth of all 4 cell lines tested (Figure 4B). Moreover, the Bliss independence model indicated synergistic effects of VIC-1911 and olaparib across all 4 cell lines, despite their differences in sensitivity to PARPi (Supplemental Table 2).

Talazoparib is another PARPi that has been recently approved as a therapeutic agent for mCRPC in combination with enzalutamide (33). We analyzed PCa cell sensitivity to talazoparib in a panel of AR-positive CRPC cells (C4-2B and 22Rv1) and AR-negative CRPC cells (DU145 and PC3) (Figure 4C). Among all 4 cell lines tested, C4-2B showed the highest sensitivity to talazoparib, and PC3 and DU145 showed a comparable sensitivity to 22Rv1 cells. Further, the IC_50_ values of talazoparib in C4-2B and 22Rv1 cells were substantially lower than those of olaparib. Since we already tested the olaparib and VIC-1911 combination in the AR-positive cell lines, here we focused on the 2 AR-negative cell lines. We observed that the combination of sublethal doses of talazoparib and VIC-1911 synergistically decreased the proliferation of both DU145 and PC3 cells, as indicated by Bliss coefficients, with a more pronounced effect in DU145 cells (Figure 4D). Saruparib, a first-in-class PARP1-specific inhibitor, was well tolerated in a phase I/II clinical study in patients with metastatic cancer (34). We sought to evaluate the effect of combining VIC-1911 with saruparib in 2 AR-dependent PCa cell lines. First, we observed that C4-2B was much more sensitive to saruparib than 22Rv1, consistent with the olaparib data (Supplemental Figure 4A). Further, VIC-1911 and saruparib cotreatment showed strong combinatorial effects in inhibiting the proliferation of both 22Rv1 and C4-2B cells. Bliss coefficient analysis suggested a synergy in 22Rv1 cells but not in C4-2B, which was already strongly inhibited by each agent alone at the dose tested (Supplemental Table 2). Taken together, our results support that VIC-1911 is a promising candidate for combination therapies with PARPi to synergistically inhibit PCa, even when HR proficient.

### VIC-1911 induces functional BRCAness, abolishing HR repair after PARP inhibition.

**Figure 5:**
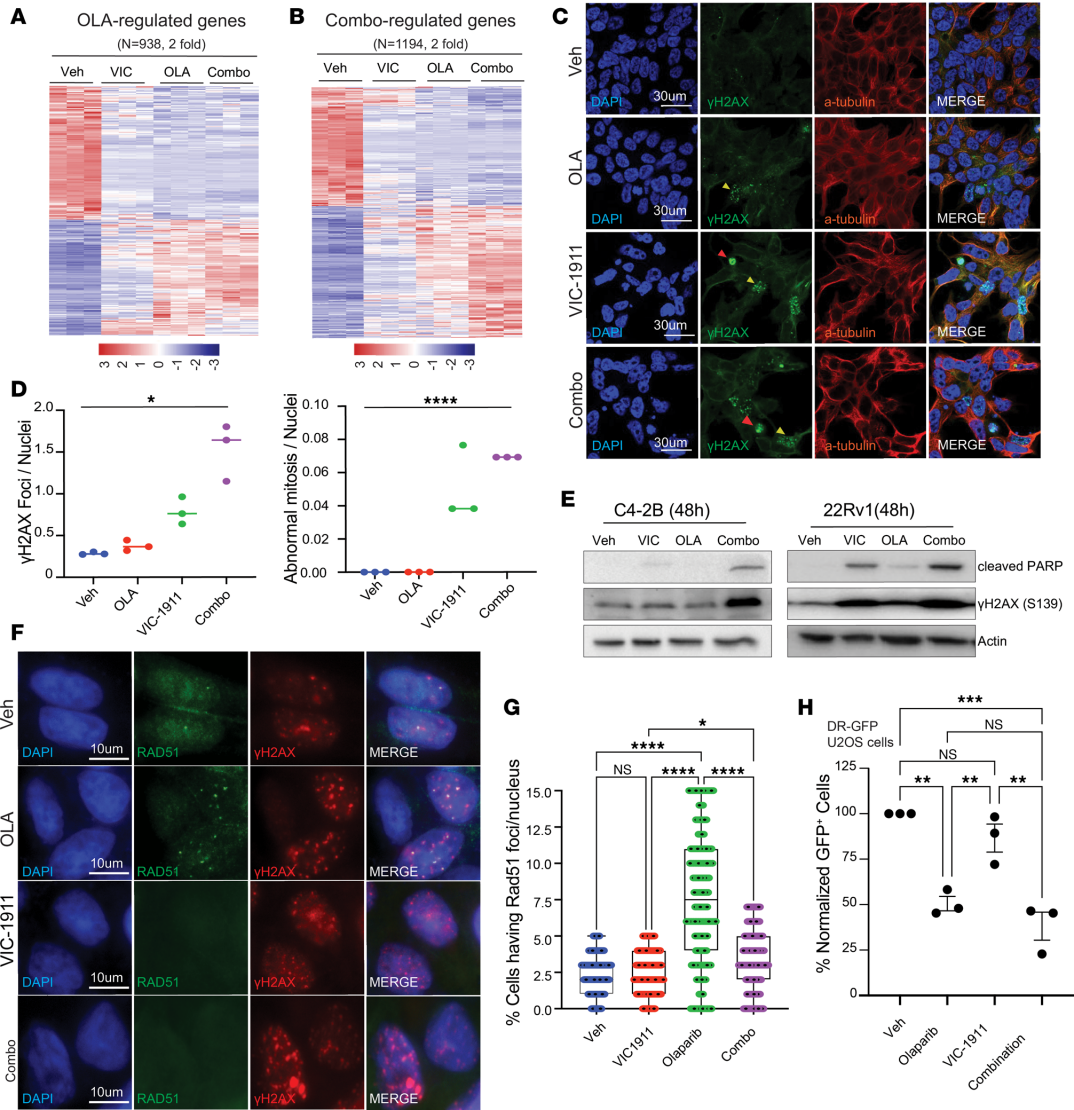
VIC-1911 induces mitotic defects and functional BRCAness, abolishing HR repair after PARP inhibition. (**A** and **B**) C4-2B cells were subjected to RNA-Seq analyses in triplicate after 7 days of treatment with vehicle or VIC-1911 (0.1 μM), olaparib (1 μM), or a combination of both. The heatmaps display upregulated and downregulated genes with a 2-fold change for olaparib and VIC-1911/PARPi combination. (**C**) VIC-1911 combined with olaparib increases the number of γH2AX nuclear foci and abnormal mitosis sites: 22Rv1 cells were treated with either vehicle, olaparib (1 μM), VIC-1911 (0.1 μM), or both for 24 hours, fixed, and subjected to confocal imaging, which revealed an increased number of nuclear γH2AX foci (yellow arrowheads) and abnormal mitotic sites (red arrowheads) in cells subjected to VIC-1911 and PARPi combination treatment. Scale bar: 30 μm. (**D**) Scatter plots show the quantification of γH2AX foci per nucleus (left) or the number of abnormal mitotic sites per nucleus (right) across 3 separate field views. Data are shown as mean ± SD, *P < 0.05, ****P < 0.0001, 1-way ANOVA combined with Dunnett’s multiple-comparison test (GraphPad Prism 10). (**E**) Immunoblotting analysis of the protein lysates derived from C4-2B or 22Rv1 cells treated with vehicle, VIC-1911 (0.1 μM), olaparib (1 μM), or a combination of both for 48 hours. (**F**) VIC-1911 in combination with olaparib inhibits olaparib-induced RAD51 foci. C4-2B cells were treated with either vehicle, olaparib (1 μM), VIC-1911 (0.1 μM), or both for 24 hours, fixed, and subjected to confocal imaging, which revealed an increased number of nuclear RAD51 foci in olaparib-treated cells, but not in cells treated with VIC-1911 or VIC-1911 and PARPi combination. Scale bar: 30 μm. (**G**) Scatter plot shows the quantification of RAD51 nuclear foci per view field; 150 fields per treatment were evaluated. *P < 0.05, **** *P* < 0.0001, 1-way ANOVA combined with Dunnett’s multiple-comparison test (GraphPad Prism 10). (**H**) Scatter plots show decreased GFP signal in DR-GFP reporter U2OS cells under olaparib (1 μM) and VIC-1911 and PARPi combination. However, no significant effect was observed under VIC-1911 (0.1 μM) treatment alone. **P < 0.01, ***P < 0.001, 1-way ANOVA combined with Dunnett’s multiple-comparison test (GraphPad Prism 10).

Next, we sought to understand the molecular mechanisms by which VIC-1911 and PARPi combination synergistically inhibited PCa. To this end, we performed RNA-Seq of C4-2B cells treated with sublethal doses of VIC-1911 (0.1 μM), olaparib (1 μM), or their combination for 7 days. We observed that VIC-1911 induced a 2-fold increase in the expression of 427 genes and a 2-fold reduction of 454 genes, olaparib induced a 2-fold increase in the expression of 434 genes and a 2-fold reduction of 504 genes, and, finally, the VIC-1911 and PARPi combination induced a 2-fold increase of 628 and a 2-fold reduction of 566 genes (Supplemental Table 3). Critically, most olaparib-repressed genes were also strongly suppressed by VIC-1911, whereas olaparib-induced genes were also slightly induced by VIC-1911, with the VIC-1911 and PARPi combination showing the strongest effect, indicating a similar molecular function (Figure 5, A and B). Indeed, GO analysis revealed that olaparib-induced or VIC-1911/PARPi combination–induced genes were strongly enriched for p53 pathways, whereas their repressed genes were involved in MTOR1 signaling (Supplemental Figure 2), being very comparable to VIC-1911–induced or –repressed genes, respectively. Together, these results suggest that VIC-1911 and olaparib regulate similar molecular processes, activating the p53 pathway while inhibiting mTORC1 signaling, which are strongly linked to DNA damage, cell cycle arrest, and apoptosis.

We subsequently asked whether the effect of the combination treatment is associated with accumulated DNA damage. To this end, we conducted fluorescent imaging to detect nuclear foci of γH2AX phosphorylation in 22Rv1 cells after overnight treatment with VIC-1911, olaparib, or their combination. We observed that olaparib alone led to some increases in phospho-γH2AX nuclear foci, but VIC-1911 as a single agent led to substantially more nuclear phospho-γH2AX, with the addition of DNA damage in cells with mitotic defects (Figure 5C). Their combination further increased the number of phospho-γH2AX foci per nucleus, indicating more severe DNA damage (Figure 5D). Moreover, there were significantly more cells with mitotic abnormalities after treatment by VIC-1911 or the drug combination. Immunoblotting analysis confirmed increased γH2AX phosphorylation and showed higher levels of cleaved PARP, a marker of apoptosis, in cells treated with VIC-1911, especially when it was combined with olaparib (Figure 5E). These results indicate that the drug combination increases mitotic defects, DNA DSBs, and apoptosis.

We next sought to determine whether the synergistic effects between VIC-1911 and PARPi are linked to altered HR activity in these cells, since PARPi is most effective in HR-deficient cancer cells. To this end, we investigated HR repair by assessing RAD51 foci formation using immunofluorescence staining in cells treated with VIC-1911, olaparib, or their combination. First, we found that olaparib alone induced a strong HR response in C4-2B cells, evident by a significant increase in nuclear RAD51 foci (Figure 5, F and G), consistent with the increase in DSBs and the HR-proficient nature of the cells. However, despite inducing more increase in DSBs, VIC-1911 did not induce nuclear RAD51 foci formation, suggesting an inhibition of HR repair. Moreover, when used in combination with olaparib, VIC-1911 diminished nuclear RAD51 foci formation expected to be induced by olaparib, manifesting a functional BRCAness phenotype. Similar results were also observed in AR-independent PC3 cells, which are HR-proficient and insensitive to PARPi (Supplemental Figure 5, C and D). These results suggest that VIC-1911 leads to functional BRCAness in PCa cells.

Next, we sought to assess whether the VIC-1911–dependent inhibition of RAD51 foci formation is due to a direct impairment of HR machinery. First, we demonstrated that this functional BRCAness was not caused by a reduction in RAD51 protein expression, as shown by immunoblotting (Supplemental Figure 5E). Next, we used U2OS cells with stable direct repeats (DR)-GFP reporter expression to assess how drug treatment affects HR repair in these cells. In the DR-GFP reporter experiment, a site-specific DSB is introduced by I-SceI endonuclease within an upstream, inactive GFP cassette. HR repair employs endogenous HR factors in these cells and a downstream truncated GFP fragment as the donor template to restore a functional GFP sequence. Successful HR repair enables GFP expression in these cells, providing a quantitative fluorescent readout (35). Interestingly, olaparib treatment decreased GFP reporter activity (Figure 5H), indicating inhibition of HR, consistent with previous reports (35). The net increase in RAD51 foci formation observed earlier in PCa cells (Figure 5, F and G) is likely due to drastically increased DSBs being repaired by an attenuated HR system. Importantly, VIC-1911 did not significantly affect HR repair of the reporter gene, despite a trend toward a decrease, in both control and olaparib-treated cells. We observed similar responses to VIC-1911 in combination with talazoparib (Supplemental Figure 5F). These findings are in contrast to its inhibition of RAD51 foci formation in PCa cells. Of note, the DR-GFP reporter system contains a downstream homologous sequence as a template for HR repair, whereas endogenous DSBs on a chromosome requires a sister chromatid as a template, the proper alignment of which, however, is likely disrupted by mitotic defects and chromosome mis-segregation upon AURKA inhibition. Therefore, by inducing mitotic defects, VIC-1911 triggers functional BRCAness, sensitizing otherwise HR-proficient PCa cells to PARP inhibition.

### VIC-1911 inhibits xenograft PCa tumor growth and sensitizes PCa to PARPi.

**Figure 6:**
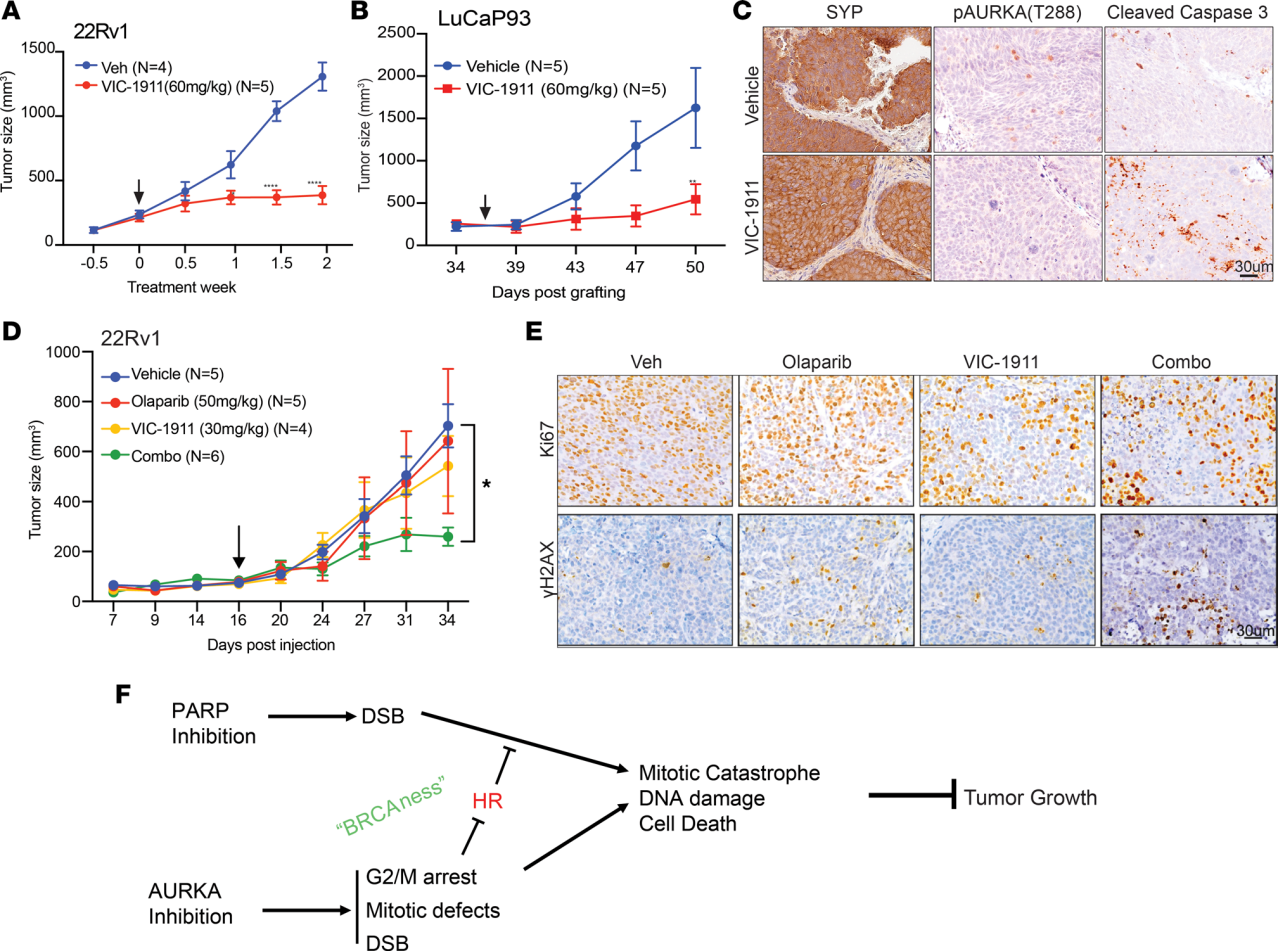
VIC-1911 significantly inhibits PCa tumor growth either as a single agent or in combination with PARPi. (**A**) VIC-1911 delays AR-positive CRPC tumor growth: 22Rv1 cells were s.c. injected into the right flank of Nod-SCID mice. Once tumors reached approximately 100 mm^3^, the mice were treated with either VIC-1911 (60 mg/kg) or vehicle for 2 weeks. Tumor growth data are shown as mean ± SEM. ****P < 0.0001, 2-way ANOVA repeated-measures test combined with Bonferroni’s multiple-comparison test. Arrow indicates starting point of treatment. (**B**) VIC-1911 delays AR-negative CRPC tumor growth. LuCaP93 patient-derived xenograft was s.c. grafted in flanks of Nod-SCID mice on both sides. Once the tumors reached approximately100 mm^3^, mice were started on treatment with either VIC-1911 (60 mg/kg) or vehicle for 2 weeks. Tumor growth data are shown as mean ± SEM. **P < 0.01, 2-way ANOVA repeated-measures test combined with Bonferroni’s multiple-comparison test. Arrow indicates starting point of treatment. (**C**) Representative IHC images confirm the reduction of phospho-AURKA in VIC-1911–treated tumors and increased staining for cleaved caspase-3, an apoptosis marker. Synaptophysin (SYP) staining validates the neuroendocrine phenotype of the AR-negative CRPC tumors of LuCaP93. Scale bar: 30 μm. (**D**) 22Rv1 (0.5 × 10^6^) cells were s.c. injected into the right flanks of Nod-SCID mice. Sixteen days after inoculation, mice were treated with either vehicle, olaparib (50 mg/kg), VIC-1911 (30 mg/kg), or a combination of both for 2 weeks. Tumor growth data are shown as mean ± SEM. *P < 0.05, 2-way ANOVA repeated-measures test combined with Bonferroni’s multiple-comparison test. (**E**) Representative IHC images of tumor sections stained for Ki67 or phospho-γH2AX (S139) are shown. Scale bar: 30 μm. (**F**) A model depicting VIC-1911 induction of functional BRCAness, sensitizing PCa cells to PARP inhibitors. VIC-1911 triggers G2/M arrest and mitotic defects, thereby compromising HR repair of DSBs induced by VIC-1911 itself and by PARPi, leading to massive accumulation of DNA damage and mitotic catastrophe and synergistically inhibiting tumor growth.

To investigate the ability of VIC-1911 to inhibit PCa tumor growth in vivo, we first evaluated its efficacy as a single agent. We grafted (s.c.) Nod-SCID mice with 22Rv1 cells, representing the majority of CRPC tumors that were AR-positive, and treated tumor-bearing mice with either vehicle or 60 mg/kg of VIC-1911 twice a day for 2 weeks. We observed that VIC-1911 significantly inhibited 22Rv1 xenograft tumor growth, flattening the growth curve after 1.5 weeks of treatment (Figure 6A). Importantly, there were no statistically significant differences in the whole-body weight of the treated mice, suggesting the tolerability of the drug in vivo (Supplemental Figure 6A). Next, we examined whether VIC-1911 could inhibit the growth of currently incurable, AR-negative CRPC tumors derived from patients. To this end, we grafted Nod-SCID mice with LuCaP93, a patient-derived xenograft of CRPC with neuroendocrine features (36). Importantly, there was a clear decrease in tumor growth after 7 days of treatment and a remarkably inhibited tumor growth within 14 days of treatment, while having no apparent effects on the whole-body weights of drug-treated mice (Figure 6B and Supplemental Figure 6B). To confirm that VIC-1911 targets AURKA, we performed histological analysis of tissue samples derived from the studied mice. IHC staining showed that VIC-1911 completely abrogated phospho-AURKA staining but greatly increased caspase-3 cleavage compared with the vehicle-treated tumors (Figure 6C). Therefore, by targeting AURKA and inducing apoptosis, VIC-1911, as a single agent, exhibits promising antitumor activity in suppressing both AR-positive 22Rv1 and AR-negative LuCaP93 patient-derived xenograft tumor growth in vivo.

We next sought to test whether VIC-1911 could be a promising therapeutic strategy to sensitize HR-proficient CRPC tumors to PARPi. To this end, we inoculated (s.c.) Nod-SCID mice with 22Rv1 cells. After the tumors reached 100 mm³, we initiated 4 treatment arms: vehicle, VIC-1911 (30 mg/kg), olaparib (50 mg/kg), or their combination. To enable us to assess the effects of the drug combination, we reduced the VIC-1911 therapeutic dose to half of the previously tested amount to avoid complete tumor killing as a single agent. We observed that this half dose of VIC-1911 alone only slightly reduced 22Rv1 xenograft tumor growth, whereas olaparib, as a single agent, did not show a clear antitumor effect in these HR-proficient cells. Remarkably, the combination of VIC-1911 and olaparib abolished tumor growth after 14 days of treatment (Figure 6D). Once more, there was no apparent effect on the body weight of the treated mice, suggesting tolerability of the drug combination (Supplemental Figure 6C). IHC analysis of endpoint 22Rv1 tumors revealed that VIC-1911, at this half dose, already led to some patches of decreased Ki67, whereas olaparib did not reduce Ki67, consistent with its lack of tumor inhibition in this HR-proficient model (Figure 6E and Supplemental Figure 6D). Despite their strong combinatorial antitumor effects, there was no further decrease in Ki67 in tumors treated with the VIC-1911 and PARPi combination. Notably, previous studies have reported that cells arrested at G2/M can remain positive for Ki67 (37), which is maximally expressed in the G2/M phase (38). Moreover, clinical evidence indicates that changes in Ki67 expression do not necessarily correlate directly with tumor response to therapy (39). Surprisingly, cleaved caspase-3 staining showed increased apoptosis in tumors treated with VIC-1911, but this effect was also not further augmented by combination with olaparib (Supplemental Figure 6E), suggesting other cell death pathways, such as necrosis and autophagy, which have been associated with mitotic catastrophe (40). Moreover, γH2AX accumulation in tumors was markedly enhanced by combination treatment, indicating severe DNA damage relative to tumors treated with either agent alone (Figure 6E), consistent with our earlier data (Figure 5, F and G) showing that VIC-1911 inhibits HR repair initiated by olaparib. Taken together, these results indicate that the drug combination synergistically inhibits PCa tumor growth primarily by inducing mitotic catastrophe and massive DNA damage. Our findings underscore the preclinical significance and safety of using VIC-1911 to sensitize PCa, including HR-proficient PCa cells, to PARP inhibitors.

## Discussion

Our study demonstrated that VIC-1911 is a highly selective and potent inhibitor of AURKA across a diverse panel of PCa cell lines, including both AR-positive and AR-negative cell lines. VIC-1911 effectively suppressed AURKA activity, as evidenced by reduced phosphorylation of both AURKA and its substrate TACC3, while sparing AURKB and AURKC even at concentrations up to 1,000-fold higher in some cells. This level of biochemical selectivity distinguishes VIC-1911 from earlier-generation Aurora kinase inhibitors, which often exhibit off-target effects and broader toxicity profiles (13). Functionally, VIC-1911 treatment resulted in dose-dependent suppression of growth across all tested PCa cell lines. AR-positive cells such as VCaP, 22Rv1, and C4-2B were particularly sensitive, with IC_50_ values below 1 μM. In contrast, AR-negative cells (PC3, DU145) exhibited reduced sensitivity despite comparable AURKA inhibition. This difference may reflect previously reported resistance of AR-negative PCa cells to G2/M arrest and mitotic checkpoint engagement, potentially due to compensatory survival pathways (23, 24). Nevertheless, the ability of VIC-1911 to inhibit colony formation and suppress growth even in these more resistant models highlights its broad antitumor potential.

Mechanistically, our transcriptomic and biochemical analyses revealed that AURKA inhibition by VIC-1911 triggers mitotic defects, DNA damage, and apoptosis. VIC-1911 activated the p53 pathway, which responds to DNA damage and may trigger cell cycle arrest, while concurrently repressing proliferative pathways, such as mTORC1. This transcriptional reprogramming was accompanied by robust phosphorylation of γH2AX, indicative of DSBs, and by activation of the DNA damage sensors ATR, ATM, and KAP1. Importantly, these molecular changes were associated with increased cleaved caspase-3 levels, suggesting commitment to apoptosis. Confocal microscopy further confirmed that VIC-1911 induces classical hallmarks of mitotic catastrophe, including prometaphase arrest and spindle abnormalities, as well as DSBs marked by γH2AX, thereby linking AURKA inhibition to lethal mitotic failure.

Clinical studies have shown that approximately one-quarter of patients with mCRPC harbor mutations in genes involved in HR repair, including *BRCA1*, *BRCA2*, *ATM*, *CHEK2*, *FANCA*, *HDAC2*, and others (19, 41, 42). A significant clinical advancement has been the demonstration that prostate tumors harboring HR mutations are susceptible to PARP inhibition (19, 33, 42). The cytotoxic effect of PARPi is believed to result, at least in part, from the trapping of the PARPi complex on single-stranded DNA, which stalls replication and leads to replication fork collapse and cell death, particularly in cells deficient in HR repair (43–45). Therapeutic strategies capable of inducing a BRCAness phenotype, therefore, hold substantial potential to expand the clinical utility of PARPi. Along these lines, our findings reveal that the selective AURKA inhibitor VIC-1911 markedly enhanced the antitumor activity of PARPi in a panel of PCa cells with diverse sensitivity to olaparib as a single agent. Mechanistically, VIC-1911 induced functional BRCAness by promoting mitotic defects and aberrant chromosome segregation, thereby compromising HR repair of DSBs in otherwise HR-proficient cells (Figure 6F). Of note, our data suggest that VIC-1911 did not directly impair the HR machinery, in contrast to previous studies done in other cancers (46). Rather, mitotic arrest can lead to chromosomal condensation, restricting access of the large HR repair machinery to the sites of DSBs, and sister-chromatid mis-segregation, compromising HR repair (47). Our data showed that the benefit of VIC-1911 and PARPi is widely applicable for various PCa cell lines, suggesting that AURKA inhibition may sensitize a broader range of tumors to PARPi-based regimens.

These observations in vitro translated robustly into efficacy in vivo. As a monotherapy, VIC-1911 significantly inhibited tumor growth in both AR-positive (22Rv1) and AR-negative (LuCaP93) xenograft models, with no detectable toxicity, as evidenced by stable body weights. Importantly, VIC-1911 effectively abrogated phospho-AURKA levels and induced cleaved caspase-3 in tumor tissues, confirming on-target activity and apoptosis induction. When combined with olaparib in a 22Rv1 xenograft model, even a reduced dose of VIC-1911, insufficient to cause tumor regression as a single agent, potentiated olaparib to significantly suppress tumor growth. This combination also led to strong γH2AX staining in tumor tissues, consistent with enhanced DNA damage being the underlying mechanism for the combinatorial effect (Figure 6F). As there were no combinatorial reductions in apoptotic markers at the endpoint tumors, we believe that tumor cells may have died via other cell death pathways in vivo, such as necrosis, consistent with TNF-α signaling being a top molecular pathway enriched by VIC-1911/PARPi combination–induced genes (Supplemental Figure 5A).

Collectively, our findings position VIC-1911 as a next-generation AURKA inhibitor with enhanced selectivity, potent antitumor activity, and a clear mechanistic basis for inducing mitotic catastrophe and massive DNA damage across diverse PCa cell types. Given the critical role of AURKA in advanced PCa, VIC-1911, as a single agent, may offer a promising therapeutic strategy targeting a wide spectrum of PCa. Our study further provides preclinical evidence for using VIC-1911 in combination with DNA repair–targeted therapies such as ATRi and ATMi in PCa. Most importantly, our study demonstrates that VIC-1911 triggers a functional BRCAness phenotype by inducing mitotic defects, thereby compromising HR and sensitizing HR-proficient PCa cells to PARPi (Figure 6F). These findings support the clinical development of the AURKA inhibitor VIC-1911 as a combination partner for PARPi, representing a promising strategy to expand the therapeutic utility of PARP inhibition in advanced PCa and potentially across additional cancer types.

## Methods

### Sex as a biological variable.

Given that PCa affects only males, the animal study utilized exclusively male mice.

### Cell culture and reagents.

C4-2B, LNCaP, 22Rv1, PC3, DU145, and VCaP cell lines were obtained from the ATCC. LAPC4 was a gift from Jiaoti Huang (Duke University, Durham, North Carolina, USA). LNCaP, C4-2B, 22Rv1, DU145, and PC3 cells were cultured in RPMI 1640 (Gibco) with 10% FBS and 1% penicillin/streptomycin. VCaP cells were cultured in DMEM (ATCC) with 10% FBS and 1% penicillin/streptomycin. LAPC4 were cultured in 10% FBS and 1% penicillin/streptomycin in IMDM (Gibco). The cells were maintained at 37°C in a humidified incubator with 5% CO_2_. Olaparib, talazoparib, and nocodazole were purchased from TargetMol, and ceralasertib (ATRi) and lartesertib (ATMi) were purchased from MedChemExpress. VIC-1911 was provided by VITRAC Therapeutics LLC. Saruparib was provided in-house. All compounds were dissolved in DMSO for cell culture studies. All cell lines were authenticated annually and routinely confirmed to be free of mycoplasma by PCR (48).

### Immunoblotting.

Total protein lysates were prepared by washing cells once with PBS, followed by lysis through 5-minute boiling in 1× SDS lysis buffer (2% SDS, 10% glycerol, 62.5 mM Tris-HCl, pH 6.8) supplemented with protease (Roche) and phosphatase (Thermo Fisher Scientific) inhibitors. Proteins (20–40 μg) were resolved in 10% SDS-PAGE gel and then transferred onto PVDF membranes (MilliporeSigma). After blocking the membranes with 5% fat-free milk to total protein or 3% BSA for phospho-protein in TBST for 1 hour at room temperature, the membranes were incubated with appropriate dilutions of specific primary antibodies in blocking buffer overnight at 4°C. After washing, the blots were incubated with HRP-conjugated secondary antibodies for 1 hour and visualized using the ECL substrate (Amersham) and a ChemiDoc imaging system (Bio-Rad).

The following antibodies were used: anti-phospho-aurora A (Thr288)/B(Thr232)/C(Thr198) (Cell Signaling Technology, 2914), anti-pTACC3 (Ser558) (Cell Signaling Technology, 8842), anti-TACC3 (Cell Signaling Technology, 8069), anti-Aurora A (Cell Signaling Technology, 91590), anti-pATM (Ser1981) (Abclonal, AP1030), anti-pKAP1(Ser824) (Cell Signaling Technology, 4127), anti-actin (Santa Cruz Biotechnology, SC-47778), anti-cleaved caspase-3 (Cell Signaling Technology, 9664S), anti-phospho-γH2AX (S139) (Abcam, ab2893), anti-cleaved PARP (Cell Signaling Technology, 5625S), anti-GAPDH (Cell Signaling Technology, 2118S), anti-p21 (Cell Signaling Technology, 2947S), anti-ERK1/2 (Santa Cruz Biotechnology, sc-398015), anti-pERK1/2 (Thr202/Tyr204) (Cell Signaling Technology, 9101), anti-pAKT (Cell Signaling Technology, 4060), anti-AKT (Cell Signaling Technology, 4691), anti-RAD51 (Calbiochem, PC130), anti-pATR (T1989) (GeneTex, GTX128145), anti-ATR (Cell Signaling Technology, 2851), and anti-Vinculin (Abcam, ab129002).

### Cell proliferation (IncuCyte), cell viability, and colony formation assays.

Cell proliferation was monitored using the IncuCyte Live-Cell Imaging System (Sartorius Biotech), following the manufacturer’s instructions. Briefly, 3,000–5,000 cells were seeded per well in a 96-well plate. The following day, drug treatments were applied as designated, and images were acquired every 2 hours until the cells reached full confluence.

Cell viability was assessed using the CellTiter-Glo Luminescent Cell Viability Assay (Promega) according to the manufacturer’s instructions. Briefly, 3,000–5,000 cells were seeded in 96-well white opaque plates. The following day, cells were treated with a serial dilution of the indicated compounds for a total of 7 days. On day 4, the treatment media was replenished with fresh media containing the corresponding drug concentrations. At the endpoint, CellTiter-Glo reagent was added to each well, and luminescence was measured using a plate reader (BioTek).

For the colony formation assay, 6,000–10,000 cells per well were seeded in a 6-well plate and treated with varying doses of VIC-1911 for 7 days. The following day, the cells were treated with varying doses of VIC-1911 and incubated for 7 days. At the endpoint, colonies were fixed with 4% paraformaldehyde, stained with 0.5% crystal violet in 25% methanol for 15 minutes, gently washed with tap water, air-dried overnight, and imaged using the ChemiDoc imaging system (Bio-Rad).

### RNA isolation and RNA-Seq.

Total RNA was isolated from cells with the NucleoSpin RNA isolation kit (Takara). ReverTra Ace qPCR RT Master Mix kit (Toyobo) was used for RNA reverse transcription. For RNA-Seq, total RNA was isolated as described above. RNA-Seq libraries were prepared from 0.5 μg high-quality DNA-free RNA using NEBNext Ultra II RNA Library Prep kit, according to the manufacturer’s instructions. The libraries passing quality control (equal size distribution of 250–400 bp, no adapter contamination peaks, no degradation peaks) were quantified using the Library Quantification kit from Illumina (Kapa Biosystems, KK4603). Libraries were pooled to a final concentration of 10 nM and sequenced paired-end using the Illumina HiSeq 4000.

### Immunofluorescence.

Cells were seeded on poly-D-lysine–coated coverslips, and the designated treatments were initiated the following day. After treatment, cells were fixed with 4% paraformaldehyde for 15 minutes at room temperature and permeabilized with 0.1% Triton X-100 for 15 minutes. After 3 PBS washes, cells were blocked with 5% BSA in PBS for 30 minutes and then incubated overnight at 4°C with primary antibodies diluted in blocking buffer in a humidified chamber. The following primary antibodies were used: anti-phospho-γH2AX (S139) (Abcam, ab2893) and anti-tubulin (Proteintech, 11224-1-AP). After 3 washes, cells were incubated with Alexa Fluor 488–conjugated or Alexa Fluor 594–conjugated secondary antibodies (Invitrogen) for 1 hour at room temperature. Nuclei were counterstained with DAPI. Coverslips were mounted using ProLong Gold Antifade reagent (Invitrogen) and imaged using a Leica Stellaris confocal laser microscope system. Images were processed using ImageJ (NIH).

For assessing RAD51 foci formation, C4-2B or PC3 cells were grown on coverslips and treated with the indicated dosages of VIC-1911 or olaparib (Selleck Chem, S1060), or a combination of both, for 24 hours. After treatment, coverslips were washed 3 times with PBS and fixed in 4% PFA at room temperature for 15 minutes, then permeabilized with 0.01% PBST (PBS with Triton X-100) for 5 minutes. Cells were washed and then blocked for 1 hour with 5% BSA at room temperature and incubated with the indicated primary antibody overnight at 4°C in a humidified condition. The next day, cells were washed 3 times for 5 minutes each using 5% BSA in PBS and then further incubated with secondary antibodies (rabbit Alexa Fluor 488, A-11008; mouse Alexa Fluor 555, A-21422 (Invitrogen) for 1 hour at 37°C. To visualize nuclei, cells were stained with DAPI and mounted on cover slides. To plot the graph showing the percentage of cells positive for RAD51 foci, each field was counted manually. Specifically, 50 fields were analyzed per experimental condition, totaling 150 fields across 3 technical replicates. Immunofluorescence signals were visualized either by a Leica SP8 inverted confocal microscope at 63× magnification or on a Zeiss Observer Z1 microscope equipped with AxioVision Rel 4.8 software.

### IHC.

IHC was performed on paraffin-embedded tissue sections. After deparaffinization and rehydration, antigen retrieval was conducted using citrate buffer (Invitrogen) according to the manufacturer’s protocol. Slides were permeabilized with 0.5% Triton X-100 and blocked using a ready-to-use Vectastain kit (Vector Laboratories) per the manufacturer’s instructions. Primary antibody incubation was performed overnight at 4°C in a humidity chamber. The following antibodies were used: anti-phospho-aurora A (Thr288) (Cell Signaling Technology, 3079), anti-synaptophysin (SYP) (Santa Cruz Biotechnology, sc-17750), anti-cleaved caspase-3 (Cell Signaling Technology, 9664S), anti-phospho-γH2AX (Ser139) (Abcam, ab2893), and anti-Ki67 (Abcam, ab16667). After 3 washes with 1× Tris-buffered saline (TBS) for 5 minutes each, detection was performed using the Vectastain ABC kit (Vector Laboratories), and signals were visualized with DAB incubation for 1–2 minutes at room temperature. Slides were counterstained with hematoxylin for 15 seconds, rinsed with tap water, dehydrated through graded ethanol washes, cleared in xylene, and mounted using Permount (Fisher Chemical). Imaging was conducted using an Olympus BX41 microscope equipped with an Olympus U-TV0.5XC-3 camera.

### DR-GFP reporter assay.

To measure HR efficacy, 50,000 U2OS cells/mL (6 cm plate, 3 mL total) stably expressing the DR-GFP reporter were transfected with 2.5 μg of the I-SceI plasmid. At 24 hours after transfection, fresh medium was added, and the next day, cells were treated with the indicated doses of olaparib, VIC-1911, or both for 24 hours. Then, the cells were washed twice with ice-cold PBS and resuspended in 500 μL PBS and subjected to flow cytometry analysis (Aurora Cytek) to measure the percentage of GFP-positive cells. The experiment was also repeated with the VIC-1911 combination with talazoparib.

### Xenograft mouse experiments.

For evaluation of VIC-1911 as a monotherapy, NSG male mice (The Jackson Laboratory; strain 005557), aged 5–8 weeks, were s.c. inoculated in the right dorsal flank with 1 × 10^6^ 22Rv1 cells suspended in a 1:1 solution of Cultrex UltiMatrix (R&D Systems) and PBS. After tumors reached approximately 100 mm^3^, tumor-bearing mice were randomized into 2 treatment arms: vehicle (0.5% hydroxypropyl methylcellulose in distilled water) or VIC-1911 (60 mg/kg). Treatments were administered orally, twice a day, every day for 2 weeks. In a separate experiment, NSG mice were s.c. grafted with LuCaP93 tumors in both dorsal flanks. After tumors on either side reached approximately 100 mm^3^, treatment was initiated using the same dosing regimen.

For evaluation of VIC-1911 in combination with PARP inhibition, NSG male mice were s.c. inoculated in the right dorsal flank with 1 × 10^6^ 22Rv1 cells. After tumors reached approximately 100 mm^3^, tumor-bearing mice were randomized into 4 treatment arms: vehicle (0.5% hydroxypropyl methylcellulose in distilled water), VIC-1911 (30 mg/kg, twice a day), olaparib (50 mg/kg, once a day), or the combination of both. Treatments were administered on a 5-day-on, 2-day-off schedule. Tumor dimensions were measured twice weekly using calipers, and volumes were calculated using the formula (length × width^2^ × 0.5). Mouse body weight was monitored weekly. At the study endpoint, mice were euthanized, and tumors were excised, fixed in 10% neutral-buffered formalin, and processed for paraffin embedding.

### Bioinformatics analysis.

RNA-Seq reads were filtered with Trimmomatic (v0.39) to remove adaptor reads. The reads were mapped to the NCBI’s human genome GRCh38 and quantified at the gene level using STAR (v1.5.2). FPKM (fragments per kilobase of transcript per million mapped reads) values were calculated by an in-house Perl script. BedGraph files were generated with Homer (v4.11). Differential gene expression analysis was performed by the R Bioconductor DESeq2 package (v3.21) with shrinkage estimation for dispersions. GSEA was performed using the R package with the gene set collection Human MSigDB (v2023.2) following the manufacturer’s instructions.

### Statistics.

One-way ANOVA followed by Dunnett’s multiple-comparison test was used to analyze data involving 4 independent groups. Two-way ANOVA with Bonferroni’s multiple-comparison correction was used for experiments involving repeated measurements across 4 groups. A *P* value less than 0.05 was considered statistically significant. All statistical analyses were performed using GraphPad Prism version 10.

### Study approval.

The animal study of the combinatorial treatment of VIC-1911 and olaparib was approved by the Emory University IACUC. Separately, a study evaluating VIC-1911 treatment as a monotherapy was approved by the IACUC office at Northwestern University.

### Data availability.

All next generation sequencing data generated in this study have been uploaded to the NCBI’s Gene Expression Omnibus (GEO GSE318076). Raw data and calculations are available in the Supporting Data Values file and from the corresponding author upon request. Analytic code is available from the corresponding author upon request.

## Author contributions

GG and JY conceived the project and designed the experiments. GG, SKR, HS, WX, QTTN, SS, and QC performed experiments and generated original figures. WX, HS, and SS performed animal experiments. JCZ, QC, and GG conducted bioinformatic and statistical analysis. TJM was included in the initial project discussions with JY and MH and provided VIC-1911. MH, SEF, and MAB provided critical insights from the clinical perspective. DSY and SKR provided guidance on DNA damage studies and saruparib. JY and GG wrote the manuscript. All authors read and commented on the manuscript.

## Conflict of interest

TM is employed at VITRAC Therapeutics LLC.

## Funding support

This work is the result of NIH funding, in whole or in part, and is subject to the NIH Public Access Policy. Through acceptance of this federal funding, the NIH has been given a right to make the work publicly available in PubMed Central.

- NIH/NCI 5 T32 CA260293-02 (to GG).
- Department of Defense PC220607 (to JY).
- Prostate Cancer Foundation Challenge Award 2017CHAL2008 (to JY).
- NIH/NCI R01CA178999, R01CA254403, R01CA301614, and U54CA274513 (to DSY).
- Department of Defense BC220744 (to DSY).
- Department of Defense LC240534 (to SKR).

## Supplementary Material

Supplemental data

Unedited blot and gel images

Supplemental table 1

Supplemental table 2

Supplemental table 3

Supporting data values

## Version 1 03/31/2026

In-Press Preview

## Version 2 05/08/2026

Electronic publication

## Version 3 05/13/2026

The correct name of the sixth author should be Que Thanh Thanh Nyugen
