# Supplementary material for: AURKA inhibitor VIC-1911 induces mitotic defects and functional BRCAness, sensitizing prostate cancer to PARP inhibition
Source: JCI Insight. 2026 Mar 31;11(9):e196665. doi: 10.1172/jci.insight.196665 (PMC13232484; doi:10.1172/jci.insight.196665)
Supplement: Supplemental data [file jciinsight-11-196665-s292.pdf]

Supplementary Materials

AURKA inhibitor VIC-1911 induces mitotic defects and functional BRCAness, sensitizing prostate cancer to PARP inhibition

Galina Gritsina[1], Sandip Kumar Rath[2], Hongshun Shi[1], Qi Chu[1], Wanqing Xie[1], Que Thanh Thanh Nguyen[1], Sambhavi Senthil[1], Thomas Myers[3], Mehmet A. Bilen[4,5], Sarah E. Fenton[6], Maha Hussain[6], David S. Yu[2,7], Jonathan C Zhao[1,5,7], Jindan Yu[1,5,7]

[1]Department of Urology, Emory University School of Medicine, Atlanta, GA, USA
[2]Department of Radiation Oncology, Emory University School of Medicine, Atlanta, GA, USA
[3]VITRAC Therapeutics LLC, Natick, MA, USA
[4]Department of Hematology and Medical Oncology, Emory University School of Medicine, Atlanta, GA, USA
[5]Department of Human Genetics, Emory University School of Medicine, Atlanta, GA, USA
[6]Robert H. Lurie Comprehensive Cancer Center, Northwestern University Feinberg School of Medicine, Chicago, IL, USA
[7]Winship Cancer Institute of Emory University, Atlanta, GA, USA

Supplementary Figures

Figure S1. VIC-1911 inhibits ERK1/2 but not AKT in PC cells
Figure S2. VIC-1911 inhibits cell growth across a diverse panel of PC cell lines.
Figure S3. VIC-1911 induces mitotic catastrophe and DNA damage in PC cells.
Figure S4. VIC-1911 enhances the anti-proliferative effects of saruparib in PC Cells.
Figure S5. VIC-1911 combination with olaparib promotes p53 pathway, mitotic arrest, and DNA damage, while inhibiting proliferative pathways and HR repair in PC.
Figure S6. VIC-1911 treatment is well-tolerated in mice, inhibits tumor growth, and induces apoptosis.

Supplementary Tables:

Supplementary Table S1. C4-2B_gene_list_2days_1.5fold_adjP0.05
Supplementary Table S2. Bliss Model
Supplementary Table S3. C4-2B_gene_list_7days_2fold_adjP0.05

Supplementary Figures

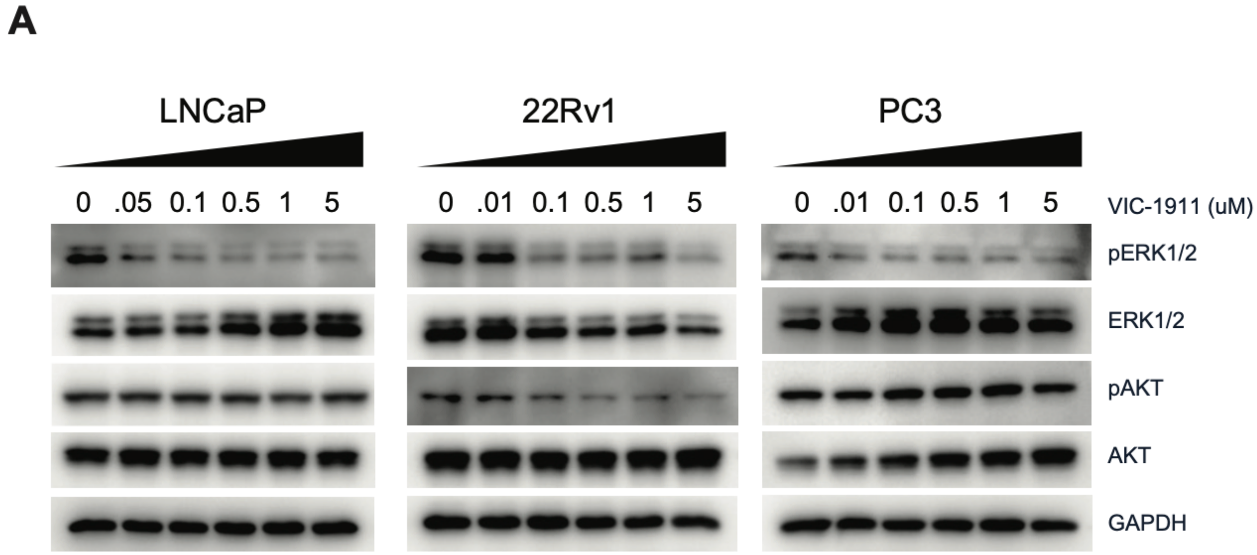

Figure S1. VIC-1911 inhibits ERK1/2 but not AKT in PC cells

(A) Immunoblotting images show a dose-dependent decrease in EKR1/2 phosphorylation under VIC-1911 treatment, while AKT phosphorylation remained largely intact. Cells were synchronized by overnight exposure to nocodazole (100 ng/ml) and co-treated with VIC-1911 for 24h.

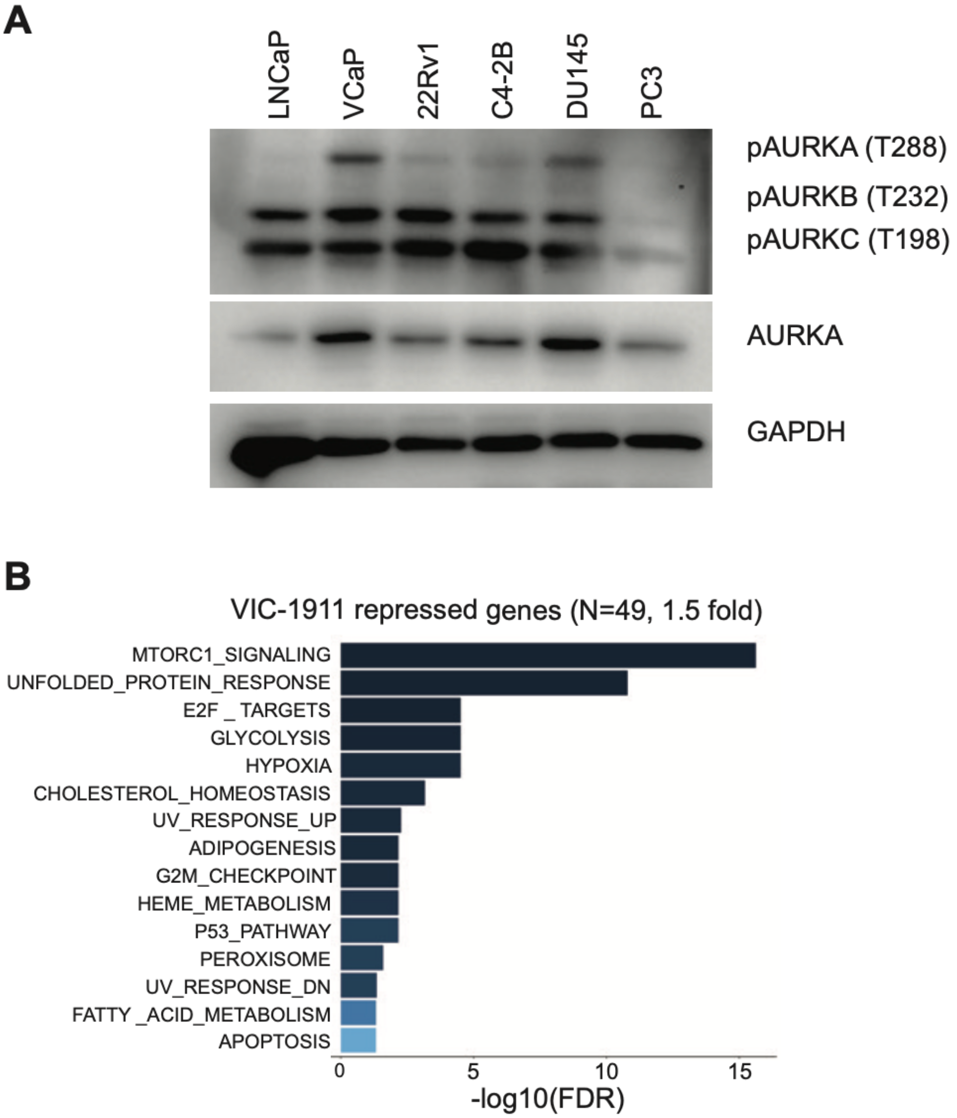

Figure S2. VIC-1911 inhibits cell growth across a diverse panel of PC
(A) Immunoblotting images demonstrate the baseline activation levels of AURKAs in unstimulated PC cells.
(B) Gene ontology research of differentially expressed genes for HALLMARK concepts that are repressed by VIC-1911 (0.1uM) treatment for 48 hours in C4-2B cells.

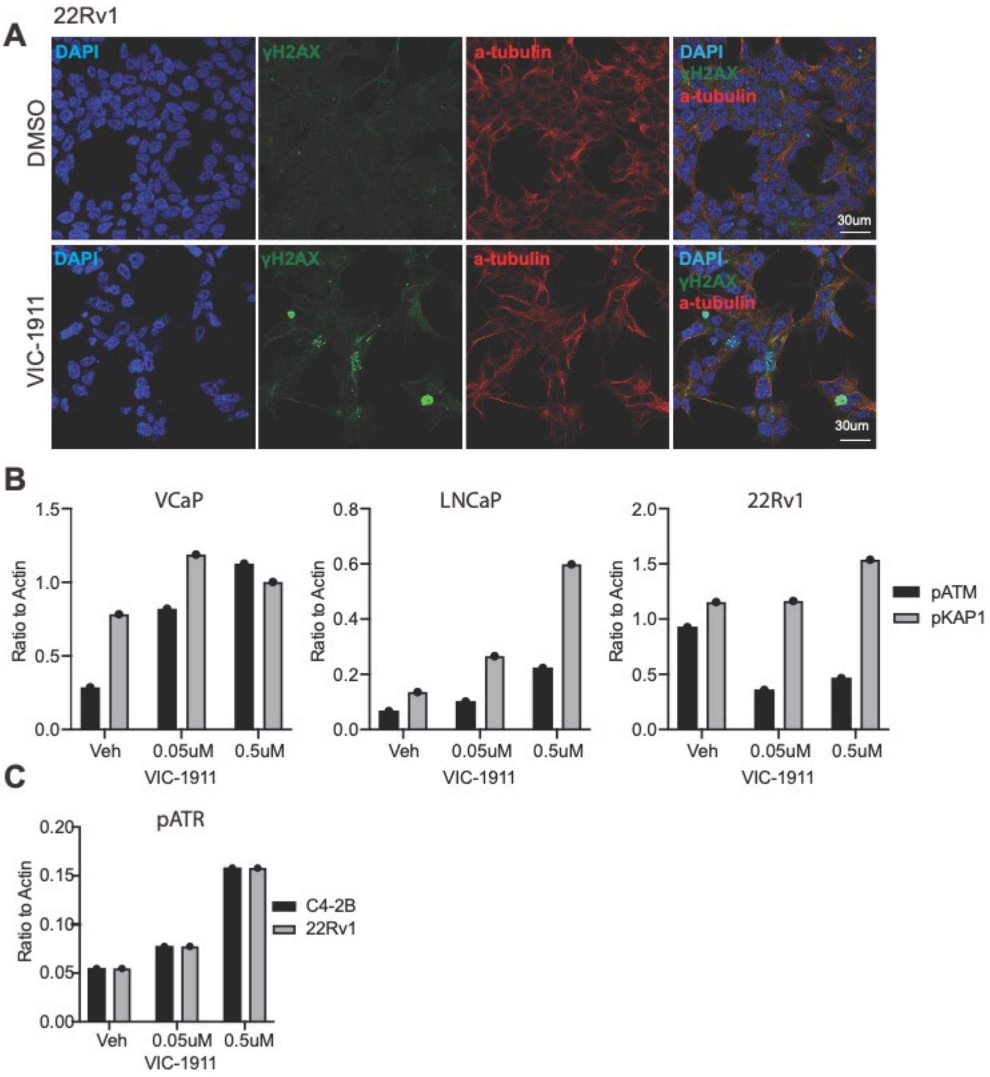

Figure S3. VIC-1911 induces mitotic catastrophe and DNA damage in PC cells.

(A) Representative immunofluorescent images show VIC-1911 induces DNA damage, evident through increased nuclear-specific phospho-γH2AX (S139) staining in 22Rv1 cells with mitotic defects after 24h of treatment with 0.1 μM of VIC-1911.

(B-C) Immunoblotting densitometry bar graphs show a dose-dependent increase in DNA damage response markers under VIC-1911 treatment. One measurement was taken**.**

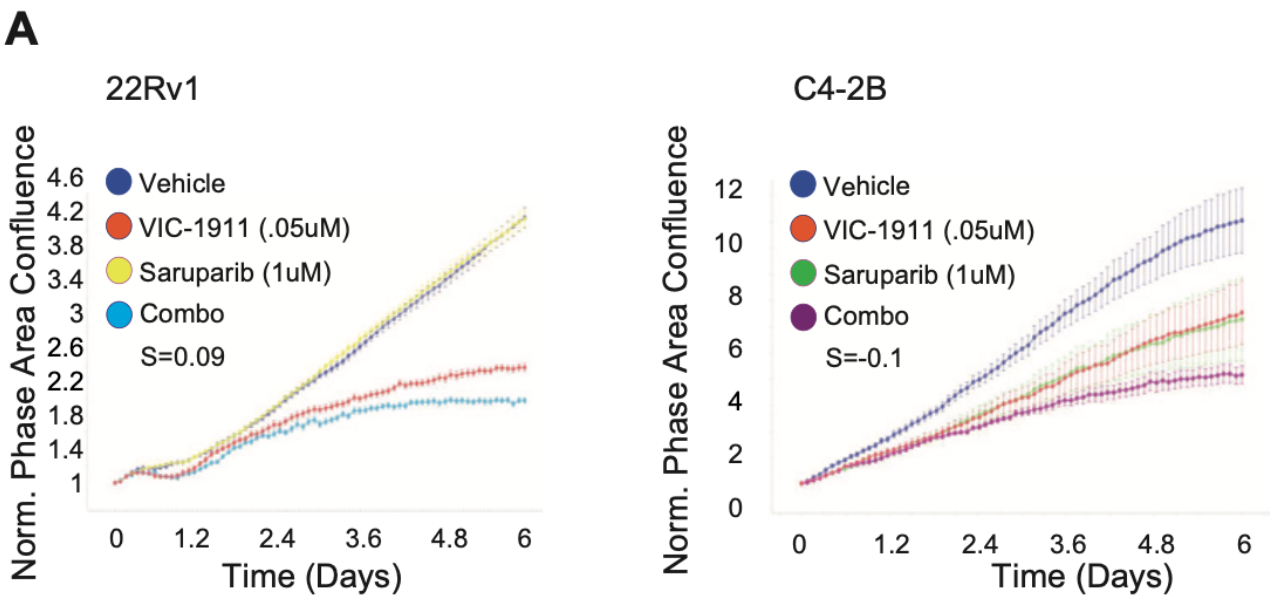

Figure S4. VIC-1911 enhances the anti-proliferative effects of saruparib in PC Cells.
(A) VIC-1911 in combination with saruparib reduces the proliferation in two AR-positive PC cells. Relative cell confluence was evaluated using IncuCyte live-cell imaging. Bliss coefficient (S): S = 0 indicates an additive effect, S > 0 indicates synergy, and S < 0 indicates antagonism. The experiment was performed once.

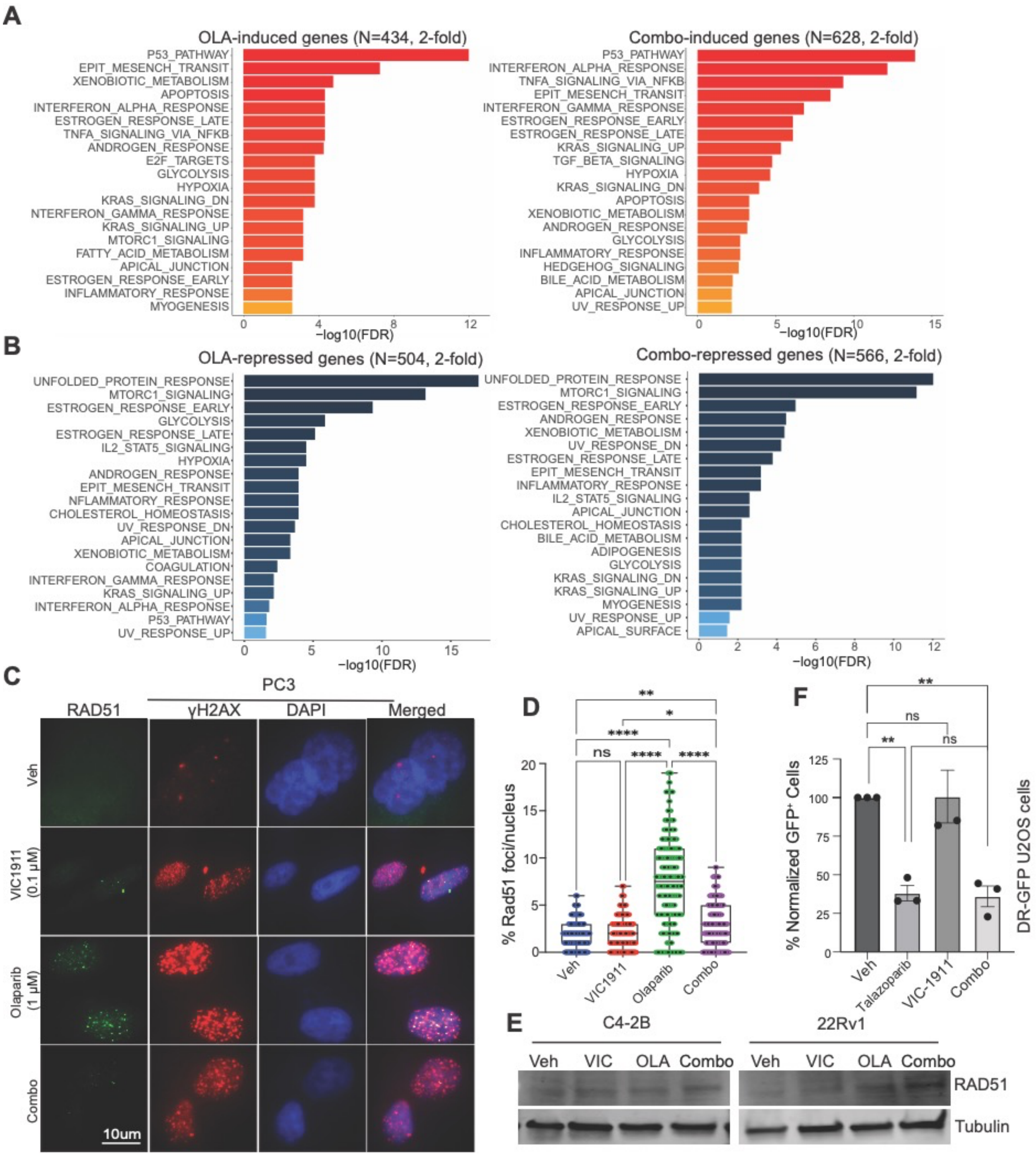

Figure S5. VIC-1911 combination with olaparib promotes p53 pathway, mitotic arrest, and DNA damage, while inhibiting proliferative pathways and HR repair in PC.

(A) Gene ontology analysis reveals a list of HALLMARK concepts that are induced by olaparib (1uM), or VIC-1911 (0.1uM) and olaparib (1uM) combo treatments after 7 days in C4-2B.

(B) Gene ontology analysis reveals a list of HALLMARK concepts that are reduced by olaparib (1uM), or VIC-1911 (0.1uM) and olaparib (1uM) combo treatments after 7 days in C4-2B.

(C) VIC-1911 in combination with olaparib inhibits olaparib-induced RAD51 foci formation. PC3 cells were treated with either vehicle, olaparib (1uM), VIC-1911 (0.1uM), or both for 24h, fixed,

and subjected to confocal imaging, which revealed an increased number of nuclear RAD51 foci in olaparib-treated cells, but not in VIC-1911 or combo-treated cells.
(D) Scatter plot shows the quantification of RAD51 nuclear foci per view field, 150 fields per treatment were evaluated. *<0.05, ****<0.0001, one-way ANOVA combined with Dunnett's multiple comparisons test (Prism 10).
(E) Immunoblotting images demonstrate the effect of VIC-1911 (0.1 uM), olaparib (1uM), or their combination on total RAD51 protein expression in PC cells.
(F) Box plot shows decreased GFP signal in DR-GFP reporter U2OS cells under talazoparib (1uM) and combo-treated cells. However, no significant effect was observed under VIC-1911 (0.1uM) treatment. **<0.01, ***<0.001, one-way ANOVA combined with Dunnett's multiple comparisons test (Prism 10).

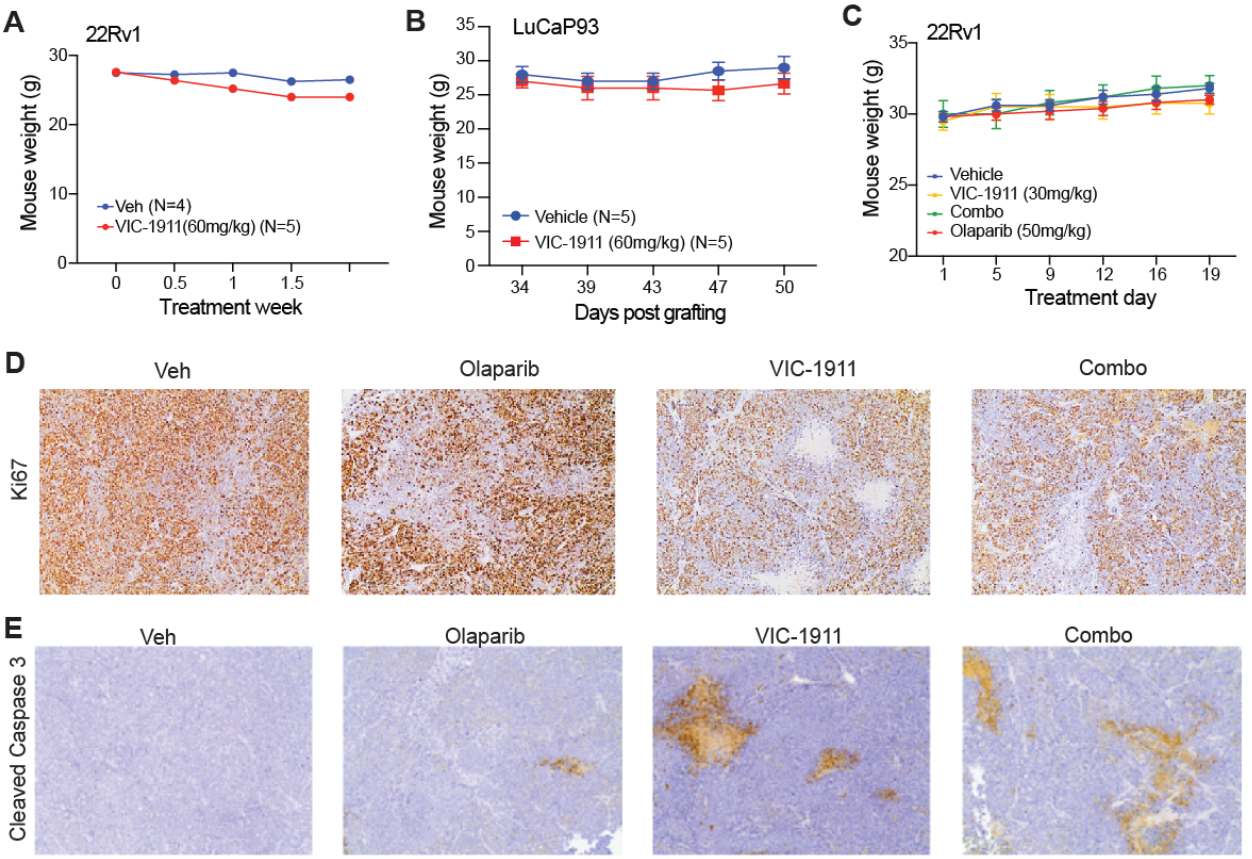

***Figure S6. VIC-1911 treatment is well-tolerated in mice*, inhibits tumor growth and induces apoptosis.**

A-C. The mouse weight charts indicate no substantial toxicity in mice s.c. inoculated with 22Rv1 and receiving VIC-1911 as a single agent (**A**), s.c. grafted with LuCaP93 and receiving VIC-1911 as a single agent **(B)**, or s.c. inoculated with 22Rv1 and treated with a combination of half the working dose of VIC-1911 and olaparib (**C**). **D-E**. IHC staining of Ki67 and cleaved caspase 3 in endpoint tumors treated with vehicle (Veh), Olaparib, VIC-1911, and their combinations.
