# Supplementary material for: AURKA inhibitor VIC-1911 induces mitotic defects and functional BRCAness, sensitizing prostate cancer to PARP inhibition
Source: JCI Insight. 2026 Mar 31;11(9):e196665. doi: 10.1172/jci.insight.196665 (PMC13232484; doi:10.1172/jci.insight.196665)

Full unedited gels for Figure 1A, 1B, 1C

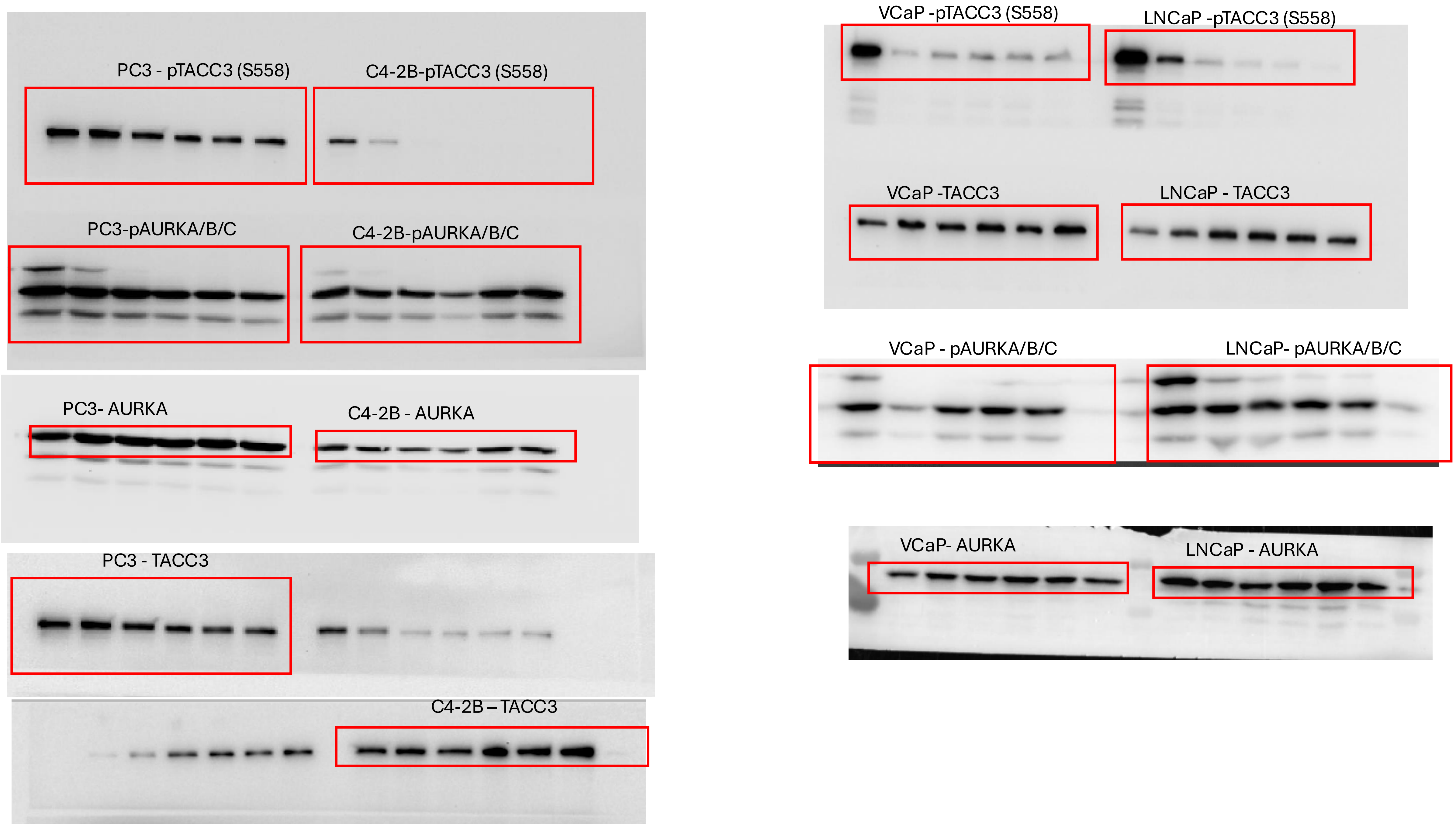

Full unedited gels for Figure 1B and 1C (Continued)

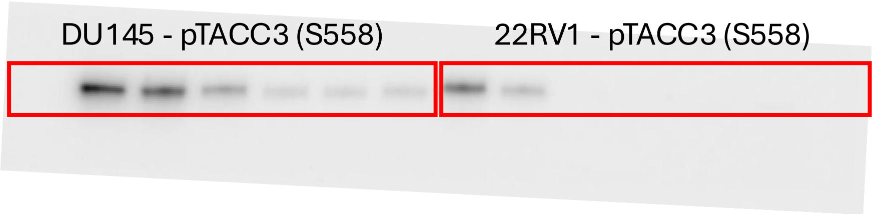

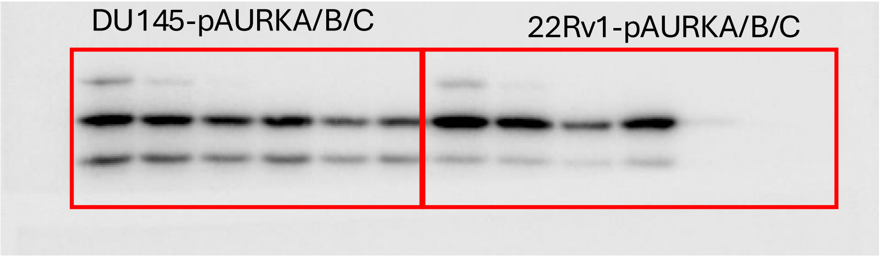

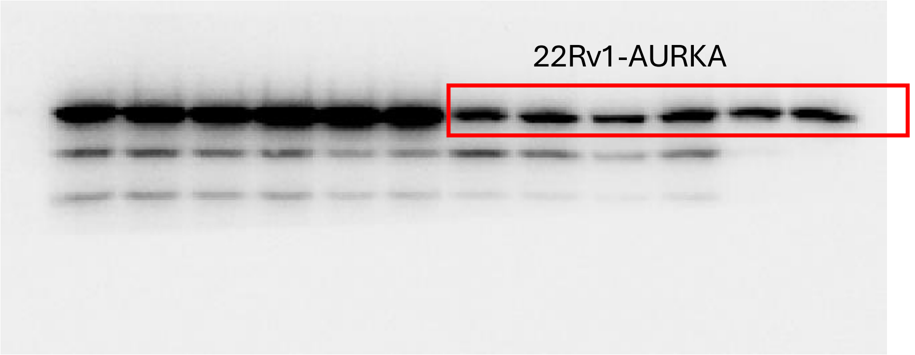

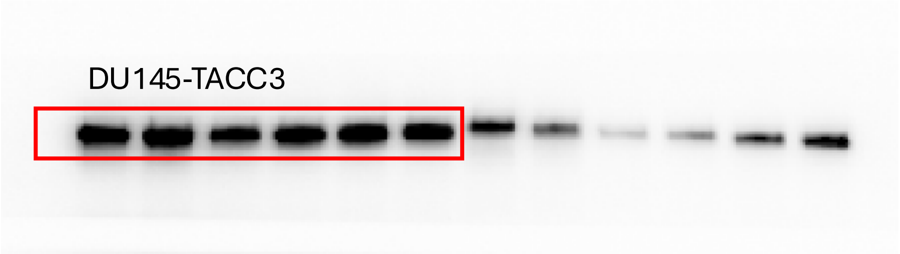

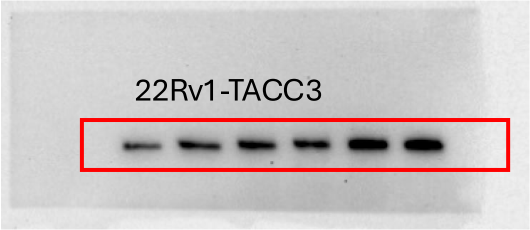

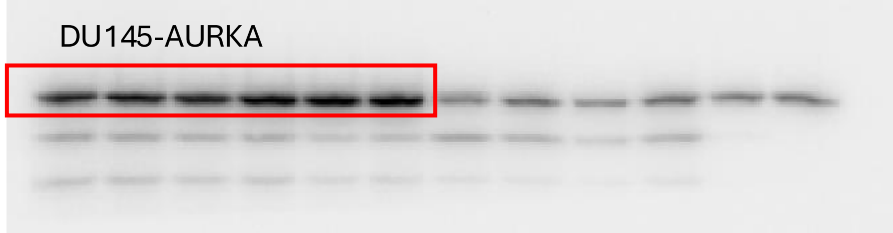

Full unedited gels for Figure 1A (Continued)

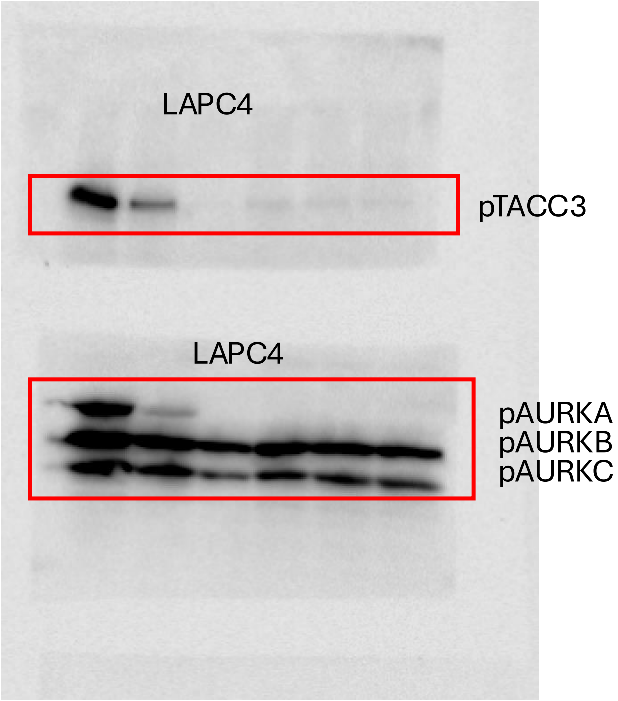

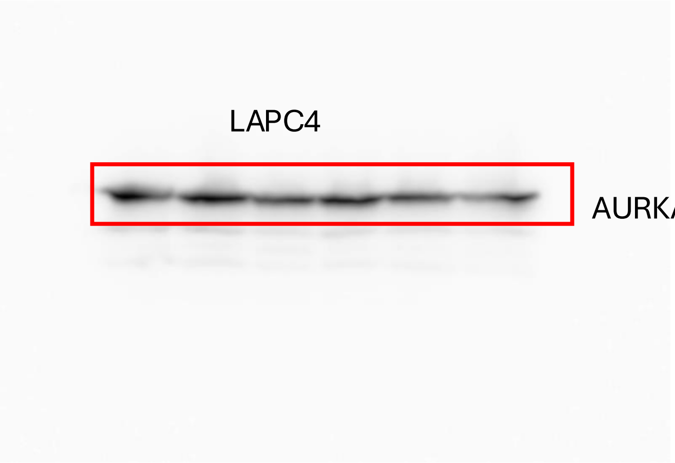

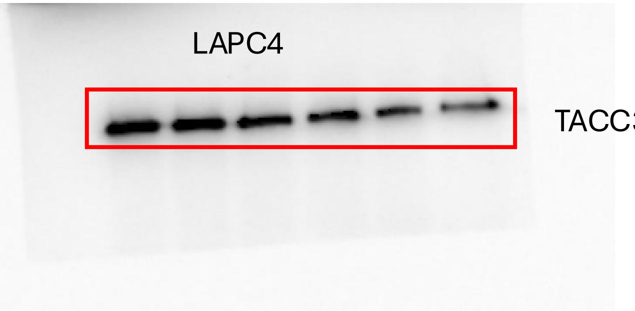

Full unedited gels for Figure 3A and 3C

C4-2B 22Rv1

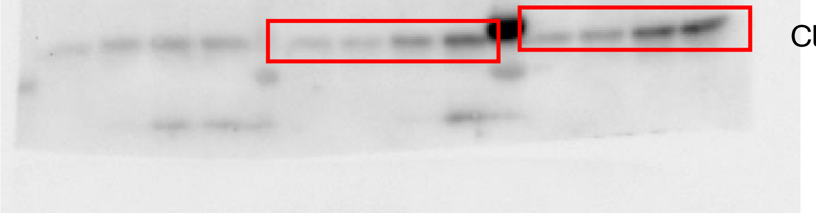

Cleaved Caspase 3

VCaP C4-2B 22Rv1

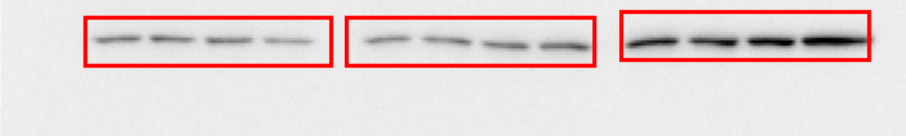

Actin

VCaP C4-2B 22Rv1

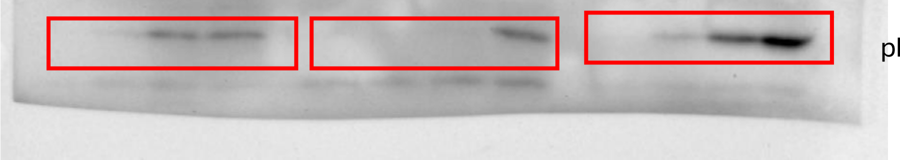

phospho-γH2AX (S139)

VCAP

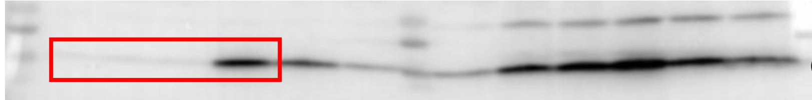

Cleaved Caspase 3

VCaP 22Rv1 LNCaP

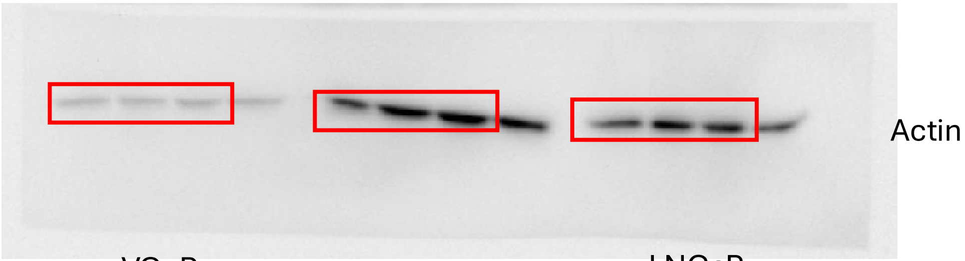

Actin

VCaP 22Rv1 LNCaP

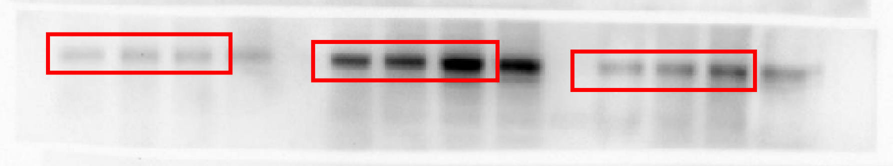

pKAP1

VCaP 22Rv1 LNCaP

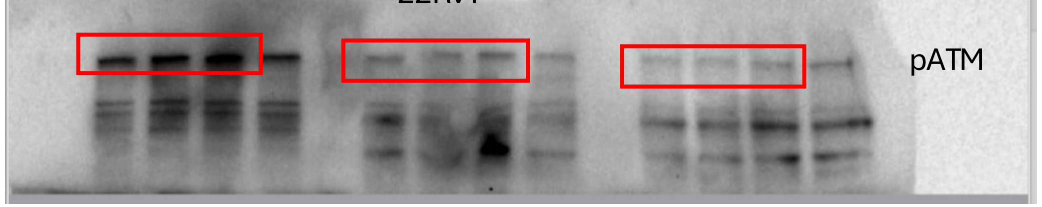

pATM

Full unedited gels for Figure 5E

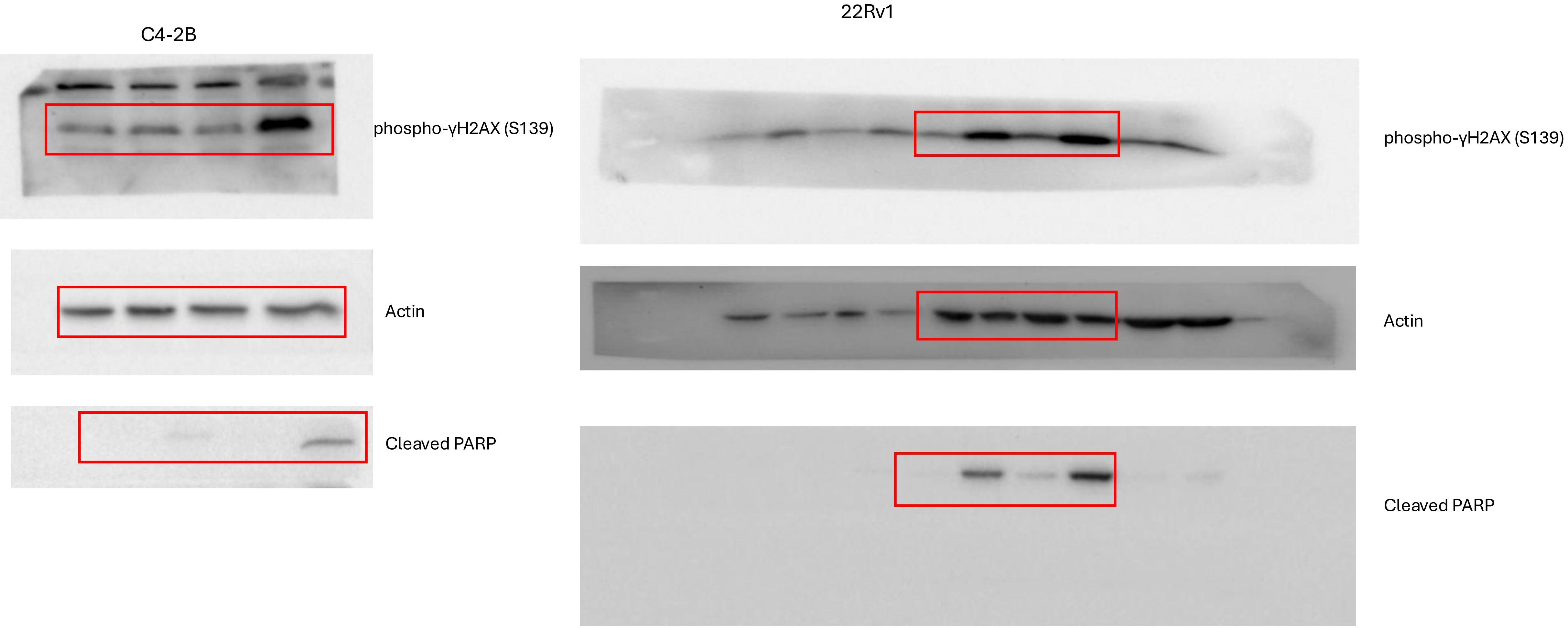

Supplement: Unedited blot and gel images [file jciinsight-11-196665-s293.pdf]
